# Lower Incidence of Dementia Following Cancer Diagnoses: Evidence from a Large Cohort and Mendelian Randomization Study

**DOI:** 10.14283/jpad.2024.135

**Published:** 2024-07-09

**Authors:** Darina T. Bassil, Bang Zheng, B. Su, D. Kafetsouli, C. Udeh-Momoh, I. Tzoulaki, S. Ahmadi-Abhari, D. C. Muller, Elio Riboli, L. T. Middleton

**Affiliations:** 1 Ageing Epidemiology (AGE) Research Unit, School of Public Health, Imperial College London, London, UK; 2 Harvard Center for Population and Development Studies, Harvard University, Cambridge, MA USA; 3 Department of Epidemiology & Biostatistics, School of Public Health, Peking University, Beijing, China; 4 Department of Epidemiology and Biostatistics, School of Public Health, Imperial College London, London, UK; 5 MRC-PHE Centre for Environment, School of Public Health, Imperial College London, London, UK; 6 Department of Hygiene and Epidemiology, University of Ioannina Medical School, Ioannina, Greece; 7 Directorate of Public Health, Imperial College Healthcare NHS Trust, London, UK

**Keywords:** Dementia, Alzheimer’s disease, cancer, competing risks, mendelian randomization analysis

## Abstract

**Background:**

The reported inverse association between cancer and subsequent Alzheimer’s disease and related dementias (ADRD) remains uncertain.

**Objectives:**

To investigate the association between these common conditions of old age and explore possible causal factors.

**Design, Setting, Participants and Measurements:**

We conducted a large population-based cohort analysis using data from 3,021,508 individuals aged 60 and over in the UK Clinical Practice Research Datalink (CPRD), over a period up to 30 years (1988–2018). Cox proportional hazards models were fitted to estimate hazard ratios (HR) for risk of dementia associated with previous cancer diagnosis. Competing risk models were employed to account for competing risk of death. Two-sample Mendelian Randomization analysis based on meta-analysis data from large-scale GWAS studies was also conducted.

**Results:**

In the CPRD cohort, 412,903 participants had cancer diagnosis and 230,558 were subsequently diagnosed with dementia over a median follow-up period of 7.9 years. Cancer survivors had a 25% lower risk of developing dementia (HR=0.75, 95% CI:0.74–0.76) after adjustment for potential confounders. Accounting for competing risk of death provided a sub-distribution HR of 0.56 (95% CI:0.55–0.56). Results were consistent for prevalent and incident cancer and different common cancer types. Two-sample Mendelian Randomization analysis, using 357 cancer-related instrumental single-nucleotide polymorphisms (SNPs) revealed evidence of vertical pleiotropy between genetically predicted cancer and reduced risk of Alzheimer’s disease (OR=0.97,95% CI:0.95–0.99).

**Conclusion:**

Our results provide strong epidemiological evidence of the inverse association between cancer and risk of ADRD and support the potential causal nature of this association via genetic instruments. Further investigations into the precise underlying biological mechanisms may reveal valuable information for new therapeutic approaches.

**Electronic Supplementary Material:**

Supplementary material is available in the online version of this article at 10.14283/jpad.2024.135.

## Introduction

**T**he distribution by age of the world’s population has been changing over the last few decades, with middle and older age groups representing a growing share of the total population. This is demographically accounted for by the combination of decreased natality and increased life expectancy. The unprecedented increase of life expectancy has resulted in an increase of the absolute number of people affected by Alzheimer’s disease and related dementias (ADRD), even if their age-specific incidence rates may have remained stable or even decreased (1).

Several recent studies, including meta-analyses, have reported an inverse association between cancer and dementia, whereby cancer survivors have a reduced risk of developing ADRD (2–13). Conversely, this inverse relationship has not been observed in other studies (14–18), some raising the hypothesis that the association, when observed, may have been due to selective mortality and competing risk of death, as individuals diagnosed with cancer might not live long enough to develop age-related dementia compared to cancer-free individuals (14,16). However, a recent simulation-based study reported that bias induced by selective survival was too small to explain the inverse association between the incidence of cancer and dementia (19). Consequently, existing data on the relationship between cancer and dementia are suggestive of an inverse association but remain inconclusive.

Unravelling the biological mechanisms that may confer risk reduction for ADRD in cancer survivors may capitalize on the wealth of accumulating knowledge in cancer biology and shed light into molecular pathways and other factors that may contribute to (or confer protection from) the development of cognitive decline and dementia in older adults. Such new knowledge may prove informative in identifying new targets and potentially lead to the discovery and development of novel classes of disease modifying therapies for ADRD.

Our aim was to examine the relationship between cancer (overall and by type) and risk of dementia, using the Clinical Practice Research Datalink (CPRD), a large-scale electronic health records (EHR) database in the United Kingdom (20). We complemented our main analysis with sensitivity and competing risk analyses, to mitigate the possible effect of survival bias in this cohort of older adults. In addition, we used genome wide association study (GWAS) data on ADRD (21) and cancer (22) to examine potential causal links and the presence of vertical versus horizontal pleiotropy through Mendelian Randomization (MR) analyses.

## Methods

### Cohort analysis on association between cancer diagnosis and risk of dementia

#### Study design and participants

Data were obtained from CPRD, an ongoing primary care database of longitudinal fully coded medical electronic health records (EHR) of over 17 million individuals, who had been registered with 718 participating general practices in the UK for different lengths of time, over the study period (20). CPRD has the advantage of being representative of the general UK population (20) and allows for a secure anonymized linkage to secondary care data of the Hospital Episode Statistics (HES) database, as well as mortality data, through the Office for National Statistics (ONS).

The observation period extended from 1988 to 2018. Cohort entry was initiated from first registration date (01/01/1988) or date at age 60, whichever was the latest. It was required that participants had been under observation for over one year prior to cohort entry, to allow time for current and past medical history information to be recorded. The end of follow-up was defined as date of dementia diagnosis, death, transfer out date, last data collection date of GP practice, or the date of 01/05/2018, whichever occurred first. Individuals with a dementia diagnosis at time of entry or prior to the age of 60 were excluded from the analyses, as they likely had familial or another early-onset dementia form. After exclusions, 3,021,508 eligible participants, aged over 60, were included in the analysis.

This cohort study has been approved by the Independent Scientific Advisory Committee (ISAC) for Medicines and Healthcare products Regulatory Agency (MHRA) database research (protocol number: 16_219R2).

#### Ascertainment of cancer cases and outcome

Cancer diagnosis was defined as the first medical diagnosis (Medcode) for cancer recorded in CPRD (Supplementary Table 1). An overall cancer category was created as a general disease variable to capture all cancer types in aggregate, including cancer codes of non-specified sites or type or primary origin, such as NOS (not otherwise specified) or metastatic cancers with unspecified primary. Individuals with diagnosis of in situ carcinomas, benign tumours or non-melanoma skin cancer were excluded. Based on current Cancer Research UK statistics on the most common cancers in the UK, the association of each of lung, breast, prostate, colorectal and melanoma skin cancer was further evaluated in relation to the risk of being subsequently diagnosed with dementia. Given the varying accuracy of recording dementia diagnosis in primary care, the diagnostic limitations of the commonest late-onset and non-familial forms of dementia in the absence of biomarker-based evidence of the pathologic Amyloid-Tau-Neurodegeneration (ATN) signature for AD, and the presence of mixed pathologies in most ADRD cases in the elderly (23–26), all cases of dementia were included in the outcome definition, with the following exclusions: dementia following HIV infection, Creutzfeldt-Jakob disease, or alcohol- and drug-induced forms. Patients were considered to have dementia if they had (1) a dementia diagnosis based on Medcode in CPRD or ICD codes in linked HES or ONS database (Supplementary Table 2), or (2) at least one dementia-specific drug prescription (donepezil, galantamine, rivastigmine, or memantine; Supplementary Table 2). The event date was defined as the first dementia diagnosis date, date of death due to dementia, or the first prescription date of dementia-specific drugs, whichever occurred first. Among the extracted dementia cases, 97% were based on diagnostic codes and 3% on prescriptions.

Information on the following potential confounders was also extracted: age, sex, calendar year, region, Index of Multiple Deprivation (IMD, a proxy variable of socioeconomic status), body mass index (BMI, <25, 25-<30, ≥30 kg/m^2^), smoking status (non-smoker, ex-smoker, current smoker), and history of diabetes and cardiovascular diseases.

#### Statistical analysis

Cox proportional hazard models, with age as the timescale, were used to determine the hazard ratios (HR) and 95% confidence intervals (CI) for risk of dementia associated with cancer (all-types in aggregate and five subtypes). Individuals were considered at risk and contributed person years of follow up from date of cohort entry to date of diagnosis of dementia, death, exit from CPRD, or end of the follow-up period, whichever came first. Cancer was treated as a time-varying covariate, such that participants who developed cancer during follow-up contributed person-years to the no cancer group up until the date of cancer diagnosis, and to the cancer group henceforth. Individuals who were diagnosed with cancer prior to cohort entry contributed person years to the cancer group. The models were adjusted for sex, region, calendar year (to control for birth cohort effect and the increasing rate of reporting dementia diagnosis in CPRD over time (27)), IMD, smoking status, BMI category, diagnosis of diabetes, and diagnosis of cardiovascular diseases. All except sex, region and IMD were modelled as time-varying covariates and updated at cancer diagnosis date for those who developed cancer during follow-up.

To account for death as a competing risk and evaluate the impact of selective mortality, Fine and Gray competing risk models were fitted to estimate subdistribution HR for dementia, adjusting for potential confounders, as above.

In secondary analysis, prevalent cancer was separated from incident cancer to examine the potential “survivor’s effect”. Participants with cancer diagnosed before cohort entry were defined as “prevalent cancer cases”; those who were diagnosed with cancer during follow-up were defined as “incident cancer cases” and those with no record of cancer diagnosis were used as the reference group. Incident cancer cases were censored at (and contributed person years up to) the date of cancer diagnosis. Additionally, separate analyses were conducted for the most common reported cancer types: lung, breast, prostate, colorectal, and skin melanoma.

As most ADRD patients develop the clinical syndrome over the age of 75, additional sensitivity analyses were conducted by restricting to: (1) individuals who were at least 70 years old at entry and had GP medical records after the age of 70; (2) individuals who entered the cohort after year 2000 to minimize potential missing data and under-diagnosis; (3) individuals who survived at least up to the age of 80 to account for selective mortality; (4) individuals who did not develop dementia within the first two years of follow-up, to minimize the risk of reverse causality; and (5) individuals without missing baseline data to take into consideration the possible incomplete control for confounding due to missing data in covariates. Stata 14 (StataCorp, College Station, Texas, USA) was used for the analysis.

### Two-sample Mendelian Randomization analysis

The cancer-related genetic instruments selected for this analysis were obtained from published GWAS studies on cancer, available in the National Human Genome Research Institute-European Bioinformatics Institute (NHGRI-EBI) GWAS catalogue, released in August 2020 (22). Summary output data on associations between genome-wide single nucleotide polymorphisms (SNPs) and each of the five cancer types were extracted from the largest GWAS studies that included participants of European ancestry. Genome-wide significant SNPs (P <5×10−8) associated with each cancer type were selected. Associations between the cancer-related SNPs and risk of Alzheimer’s disease were obtained using GWAS meta-analysis data from the International Genomics of Alzheimer’s Project (IGAP) 2019 report (21), including data from 21,982 AD cases and 41,944 controls of European ancestry.

Firstly, linkage disequilibrium-based SNP pruning was performed to ensure that the included SNPs for each cancer type are independent genetic variants (with a threshold of r2<0.1).1 If a particular exposure SNP (cancer-related) was not present in the IGAP dataset, available proxy SNPs in strong linkage disequilibrium (r2>0.8) were used instead (28).

For each selected cancer-related instrumental SNP, the SNP-AD association estimate (βAD, the logarithm of odds ratio) was divided by the SNP-cancer association estimate (βcancer) to obtain a Wald ratio, as an estimate of causal effect of cancer on AD risk when the Mendelian Randomization assumptions hold (28). As such, every instrumental SNP could provide one cancer-AD causal estimate. For each cancer subtype, inverse variance weighted method (Supplemental Figure 1) was used to synthesize the causal estimates between cancer and risk of AD obtained from all SNP instruments for that cancer subtype (29). The causal link between overall cancer and risk of AD was also estimated by combining all eligible SNP instruments for these specific types of cancer.

Furthermore, Q test was performed to assess the degree of heterogeneity of causal estimates determined from different SNPs. MR-Egger regression, funnel plot, and the MR pleiotropy residual sum and outlier (MR-PRESSO) test were conducted to assess a potential directional horizontal pleiotropy (30, 31). To further account for potential violation of MR assumptions, sensitivity analyses were conducted using MR-Egger regression, maximum likelihood method, weighted median method, and weighted mode method for parameter estimation (28).

The two-sample Mendelian randomization analysis was performed based on the MR-Base platform (www.mrbase.org) (28) and R packages (“TwoSampleMR”, “MRInstruments”, and “MR-PRESSO”).

## Results

### CPRD cohort analysis

A total of 3,021,508 CPRD participants, aged 60 and over, were included in the analysis. There were 125,666 prevalent and 287,237 incident cancer cases in this cohort, whilst 230,558 incident dementia cases were observed during a mean follow-up period of 9.3 years (median 7.9 years) corresponding to 28,227,387 person-years between 1988 and 2018. Compared to individuals without cancer at baseline, those with prevalent cancer were slightly older, had lower BMI, more likely to be female, of lower Index of Multiple Deprivation (i.e., more deprived), former smokers, and have a diagnosis of diabetes and cardiovascular diseases (Table 1).

**Table 1:** Baseline characteristics of participants with and without cancer diagnoses at time of cohort entry (N=3,021,508)

**Baseline characteristics**	**No cancer diagnosis at baseline (n=2,895,842)**	**Prevalent cancer (n=125,666)**
Age (mean±SD), years	65.7 ± 8.3	68.0 ± 9.7
Male, %	46.1	39.2
Year of cohort entry (median)	2000	2004
IMD level, %		
1 (most deprived)	22.3	24.8
2	19.5	20.0
3	21.5	21.9
4	20.5	19.1
5 (least deprived)	16.2	14.2
Smoking status, %		
Non-smoker	60.4	56.2
Former smoker	20.2	26.2
Current smoker	19.4	17.6
BMI, %		
Normal/underweight (<25 kg/m^2^)	33.1	36.6
Overweight (25-<30 kg/m^2^)	40.3	38.2
Obesity (≥30 kg/m^2^)	26.6	25.2
Prevalent diabetes, %	6.0	8.7
Prevalent CVD, %	14.8	23.4

Abbreviations: SD=standard deviation; IMD=index of multiple deprivation; BMI=body mass index, CVD=cardiovascular diseases.

**Figure 1:**
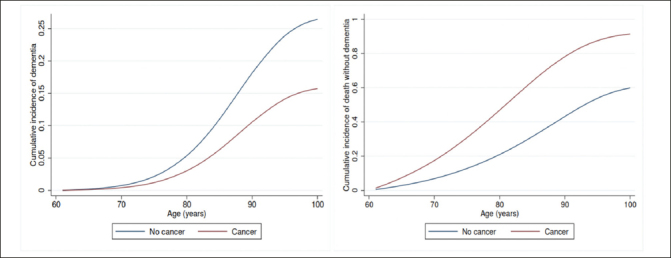
Cumulative incidence curves for dementia (left) and death (right) by cancer status after accounting for competing risks, based on Fine and Gray model

Having a diagnosis of cancer was associated with a lower risk of subsequent dementia diagnosis. Adjusted for potential confounders, the Cox proportional HR (95% CI) of dementia was 0.75 (0.74–0.76) for all cancers (prevalent or incident), 0.84 (0.82–0.85) for prevalent cancer, and 0.72 (0.71–0.73) for incident cancer in separate analyses, with the inverse association being stronger for incident cancer (Table 2). After accounting for the competing risk of death, the sub-distribution HR (95% CI) of dementia was 0.56 (0.55–0.56) for all cancers (Table 2 and Figure 1); 0.73 (0.71–0.74) for prevalent cancers and 0.48 (0.47–0.49) for incident cancers. Cancer was associated with a higher cumulative mortality rate (Table 2 and Figure 1), with sub-distribution HR (95% CI) of death without dementia being 2.69 (2.67–2.71), 1.77 (1.75–1.79) and 3.26 (3.23–3.29) for all cancers, prevalent and incident cancers, respectively.

**Table 2:** Association of overall cancer, prevalent cancer*, and incident cancer* with risk of dementia based on time-varying Cox model and Fine and Gray competing risk model (N=3,021,508)

**Models (age as time scale)**	**Overall cancer**	**Prevalent cancer (n=125,666)**	**Incident cancer (n=287,237)**
Time-varying Cox model, HR (95% CI)			
Model 1: adjust for gender, calendar year, region	0.78 (0.77–0.79)	0.87 (0.85–0.88)	0.68 (0.67–0.69)
Model 2: model 1 + IMD, smoking status, BMI category, diabetes, CVD	0.75 (0.74–0.76)	0.84 (0.82–0.85)	0.72 (0.71–0.73)
Fine and Gray competing risk model**			
sub-distribution HR (95% CI) for dementia:			
Model 1: adjust for gender, calendar year, region	0.57 (0.56–0.58)	0.74 (0.72–0.76)	0.49 (0.48–0.50)
Model 2: model 1 + IMD, smoking status, BMI category, diabetes, CVD	0.56 (0.55–0.56)	0.73 (0.71–0.74)	0.48 (0.47–0.49)
sub-distribution HR (95% CI) for death without dementia:			
Model 1: adjust for gender, calendar year, region	2.73 (2.72–2.75)	1.82 (1.80–1.84)	3.31 (3.28–3.34)
Model 2: model 1 + IMD, smoking status, BMI category, diabetes, CVD	2.69 (2.67–2.71)	1.77 (1.75–1.79)	3.26 (3.23–3.29)

Note: Numbers of observed incident dementia cases were 27,527 for cancer group (2,144,503 person-years) and 203,031 for non-cancer group (26,082,884 person-years). Non-cancer group was used as the reference group in all models. Abbreviations: HR = hazard ratio; CI=confidence interval; IMD=index of multiple deprivation; BMI=body mass index; CVD=cardiovascular diseases. * Prevalent cancer defined as diagnosis of cancer prior to study entry, incident cancer defined as cancer diagnosis over the course of follow-up. ** Sub-distribution HRs are adjusted for the competing risk of mortality.

In terms of cancer types there were 60,517 breast, 53,731 prostate, 35,601 lung, 47,966 colorectal and 15,514 melanoma cancer cases. After adjustment for potential confounding factors, all five cancer subtypes were consistently associated with lower dementia risk (Cox proportional HRs ranged from 0.64 to 0.80, P<0.05; Table 3).

**Table 3:** Association between specific types of cancer (prevalent and incident cancer combined) and risk of dementia

**Cancer types**	**Cohort participants at baseline**	**Prevalent and incident cancer cases**	**Incident dementia cases**	**Time-varying Cox model, HR (95% CI)**	
				**Model 1**	**Model 2**
Breast (female)	1,586,566	60,517	136,897	0.82 (0.80–0.85)	0.80 (0.77–0.82)
Prostate (male)	1,341,987	53,731	74,016	0.68 (0.66–0.70)	0.68 (0.65–0.70)
Lung	2,892,622	35,601	203,700	0.71 (0.65–0.76)	0.64 (0.59–0.69)
Colorectal	2,904,341	47,966	206,264	0.72 (0.70–0.75)	0.70 (0.67–0.72)
Melanoma	2,901,767	15,514	204,606	0.79 (0.75–0.83)	0.77 (0.73–0.81)

Note: Non-cancer group was used as the reference group in Cox models. Model 1: adjust for gender, calendar year (time-varying), region. Model 2: model 1 + IMD, smoking status, BMI category, diabetes and cardiovascular diseases. Abbreviations: HR = hazard ratio; CI=confidence interval.

The results of the sensitivity analyses were consistent with those of the main analysis. The Cox proportional HR (95% CI) of dementia associated with cancer was 0.77 (0.76–0.78) in analysis restricted to individuals aged over 70 at cohort entry, 0.86 (0.84–0.89) for those who entered cohort after year 2000, 0.78 (0.77–0.79) among individuals who survived up to the age of 80, 0.74 (0.73–0.75) for those who did not develop dementia within the first two years of follow-up, and 0.80 (0.78–0.82) in the analysis restricted to study participants with no missing data (Supplementary Table 3).

### Two-sample Mendelian Randomization analysis

Based on the five largest published GWAS studies (32–36) and inclusion criteria for instrumental SNPs, 172 eligible instrumental SNPs were included for breast cancer (32), 103 SNPs for prostate cancer (33), 14 SNPs for lung cancer (34), 73 SNPs for colorectal cancer (35), and 63 SNPs for melanoma (36) (all eligible SNPs are listed in Supplementary Figure 1).

**Figure 2:**
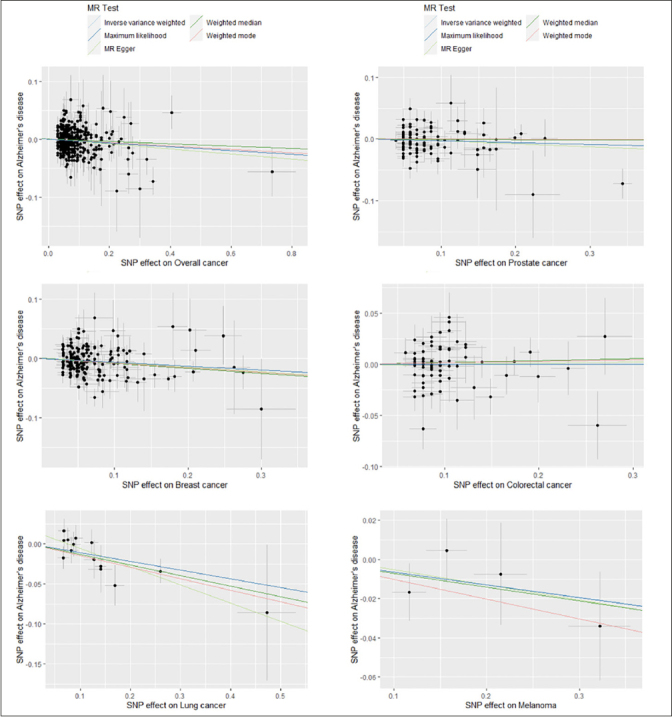
Scatter plots for estimation of causal effects of overall cancer and five cancer subtypes on risk of Alzheimer’s disease in Mendelian Randomization analysis Note: Each scatter plot displays the relationship of SNP effects on overall cancer or a specific type of cancer (x axis) against the SNP effects on Alzheimer’s disease (y axis). For each plot, one dot represents an instrumental SNP for overall cancer or a cancer subtype; five solid lines are drawn for different causal estimation methods, with the slope of the line representing the estimated causal effect.

Weighted average odds ratio (95% CI) of AD for genetically predicted breast and lung cancer were 0.94 (0.90–0.98) and 0.90 (0.84–0.96) respectively (Table 4, Figure 2, Supplementary Figure 1). Evidence of association was weaker for prostate (0.97 (0.93–1.01)), colorectal cancer (1.00 (0.96–1.04)), and melanoma (0.98 (0.94–1.02)). After combining all the instrumental SNPs of the included types of cancer, a significant inverse association was found between overall cancer (the five cancer types combined) and risk of AD (0.97 (0.95–0.99)).

**Table 4:** Mendelian Randomization analysis for causal links between overall cancer and cancer subtypes and risk of Alzheimer’s disease

**Exposure**	**Number of SNPs**	**OR (95% CI)**	**P value**	**I2 for heterogeneity**
Breast cancer	172	0.94 (0.90–0.98)	0.002	10.4%
Prostate cancer	103	0.97 (0.93–1.01)	0.183	14.7%
Lung cancer	14	0.90 (0.84–0.96)	0.003	0%
Colorectal cancer	73	1.00 (0.96–1.04)	0.995	4.5%
Melanoma	4	0.93 (0.86–1.01)	0.074	0%
Overall cancer	357	0.97 (0.95–0.99)	0.003	8.4%

Note: OR represents the odds ratio of Alzheimer’s disease associated with genetically predicted cancer (per 1-unit higher log odds of cancer). Abbreviations: SNPs = single-nucleotide polymorphisms; OR = odds ratio; CI = confidence interval.

There was no substantial heterogeneity across multiple estimates obtained from different SNPs for all five types of cancer and overall cancer (I2 ranged from 0% to 14.7%). To validate that the identified causal links between cancer and AD risk were indeed of vertical pleiotropy and not due to directional horizontal pleiotropy of the instrumental SNPs, several additional analyses were conducted (30, 31). The testing of intercept in MR-Egger regression and visual inspection of funnel plots for each cancer subtype and overall cancer showed no evidence of directional horizontal pleiotropy, with P>0.10 for all MR-Egger intercepts and no signs of funnel plot asymmetry (Supplementary Figure 2). Furthermore, the MR-PRESSO global test indicated no significant directional horizontal pleiotropy in the MR analyses of five cancer subtypes and overall cancer (P>0.10). Results of sensitivity analyses using MR-Egger regression, maximum likelihood method, weighted median method, and weighted mode method for parameter estimation revealed similar causal estimates for specific types of cancer and overall cancer to that of the main analysis (Figure 2, Supplementary Table 4).

## Discussion

In this longitudinal retrospective cohort study of 3,021,508 participants, individuals with cancer had a 25% lower hazard of developing dementia compared to individuals without a cancer diagnosis. An inverse association between cancer and risk of dementia was observed for the most common cancer types (lung, breast, prostate, colorectal and skin cancer), for both prevalent and incident cancers. Further competing risk analysis and sensitivity analyses after restricting to individuals who were at least 70 years old at cohort entry or those who survived up to the age of 80 suggest that selective mortality cannot explain the observed inverse associations. Additionally, the MR analysis results support the causality of the inverse associations between overall cancer, breast cancer and lung cancer with risk of ADRD.

In our study, the inverse association remained unaffected after treating cancer as time-varying covariate and accounting for the competing risk of death, contrary to a previous report based on a smaller cohort of 92,425 individuals (16). A recent study, based on a French cohort of memory clinic patients, with mild cognitive decline or isolated cognitive subjective complaints, showed a 50% reduction in transitioning to dementia following incident cancer (12). Another recent study, using Danish health registries, also reported an inverse association between AD and cancer but diminishing with length of follow-up (10). Similarly, a cohort study including 263,151 participants in the UK Biobank found that individuals with prevalent cancer had a lower risk of developing dementia and its subtypes, after adjusting for age and sex (13). Interestingly, a more recent study using the UK Biobank data reported contrasting findings, revealing a positive association between incident cancer and all-cause dementia, suggesting a potential diagnostic bias for dementia, particularly within the first year after cancer diagnosis (18). Another recent study revealed evidence on an inverse association between cancer and neuropathologically confirmed Alzheimer’s disease (37), with individuals with a history of cancer diagnosis exhibiting lower burden of neurofibrillary tangles and amyloid plaques (neuritic and diffuse).

The results of our two-sample MR analyses reinforce the evidence for causality of the inverse association, as previously reported (38), whilst mitigating the risk of putative confounding and reverse-causality bias. Most studies investigating the genetic association between cancer and AD have focused on the dysregulation of shared genes and pathways involved in carcinogenesis (39) that may also lower the risk of cognitive decline and dementia (40). Two GWAS-based studies revealed a significant genetic correlation between AD and overall cancer (41) and breast or prostate cancer (42), signifying a potential horizontal pleiotropic effect for these two phenotypes. While such studies may explain the correlation between two diseases, they cannot infer the presence of an independent association or causal link (43) of dementia risk following the occurrence of cancer. Our MR findings provided an additional perspective on the causal links between cancer and AD, showing evidence of a vertical pleiotropic effect. Thus, the mechanism(s) underpinning the observed negative association may be driven by biological phenomena occurring post initial cancer diagnosis.

Potential mechanisms that may explain a causal link between cancer diagnosis and reduced risk of ADRD may involve cancer-related biological phenomena that may confer neuro-protective effects. Alternatively, pharmacological treatments for common cancers may have a biological effect on the ADRD pathogenic mechanisms through immune mediated or other pathways. Several biological cancer hallmarks (44, 45) overlap with AD pathogenesis (46) and may, indeed, confer a neuroprotective effect for ADRD; these include increased cell cycles, apoptotic resistance, and evading growth suppressors, evading (or increased repair of) genomic instability and damage, as well as immune “escaping” mechanisms (47) and systemic inflammation (48). PIN1 and p53 have been proposed as potential key molecular players in the inverse cancer-AD association; specifically, PIN1 reported to contribute to cancer initiation and progression has been shown to catalyze the cis-to-trans isomerization of both amyloid precursor protein (APP) and tau, and thus reduce the accumulation of amyloid and hyper-phosphorylated tau involved in neurodegeneration (49). Furthermore, the observed inverse association between cancer and dementia risk may add credence to the potential repurposing of anticancer drugs for dementia prevention (2, 50). However, evidence on efficacy of specific chemotherapies on dementia incidence or progression remains sparse and inconclusive (49). Moreover, cognitive impairment is a known adverse effect of cancer chemotherapies, termed as “chemobrain” or “chemofog” (51, 52). Emerging evidence suggests that this may be mediated through a drug-induced tri-glial (microglia, astrocytes, and oligodendrocyte lineage cells) dysfunction (53), but the complex interplay between glia and neurons and resulting neurotoxicity are not fully understood (54, 55). As the optimal timing for disease modifying intervention in ADRD is within the at risk pre-symptomatic or early symptomatic stages, safety represents a major consideration for drug repurposing in this vulnerable population of older adults. The emergence of new generation immunotherapies (56) and other novel anticancer agents with a better safety profile may create a pool of potentially effective candidate drugs for ADRD.

The key strengths of this study lie in its sample size of 412,903 cancer and 230,558 dementia cases diagnosed in a cohort of over 3 million participants, long observation period of up to 30 years, large age distribution of the population under study, and the use of genetic data for identifying causal associations. We were also able to control for selective mortality, using thorough statistical model specification and competing risk analysis, which represent imperative steps in epidemiological research in the older adult population. In this study, besides the overall cancer diagnosis, we also conducted specific analyses on the five most common cancers in the UK (based on the Cancer Research UK statistics); future studies on other prevalent cancer types such as hematological cancer may provide further insights on the cancer-dementia link.

Several limitations should be considered when interpreting our results. These include the potential under-reporting of dementia in CPRD, for which we attempted to mitigate by adjusting for calendar year and in using linkage to secondary care and mortality databases to identify dementia cases that may have not been recorded by primary care physicians. Furthermore, the diagnostic classification of late-onset dementias has (until very recently) been problematic in the absence of biomarker-based evidence of the underlying neuropathology. Hence, the term of ADRD has been employed in this study, as shown to be inclusive of late-onset dementia cases, with AD representing over 90% of ADRD (57). Given the long pre-clinical stages associated with progressively accumulating pathology and insidious clinical onset that are typical of ADRD, it is possible that study participants may have had early mild cognitive decline, which was not recorded in a timely manner due to failure in recognizing their symptoms and in seeking medical advice. Equally, dementia patients may have harboured cancer pathology that had remained undiagnosed prior to dementia diagnosis. Selection bias is another inherent risk of real-world EHR-based data. In our study, this risk was mitigated by the use of the UK national primary care database including data from virtually all individuals registered in 8% of GP practices, covering both rural and urban communities of diverse socio-economic strata and shown to be well representative of the UK general population (20). Missing data is an acknowledged challenge of large-scale EHR-based studies (20). We had significant missing baseline values for IMD, smoking status and BMI, with only slight difference of effect estimates in models with and without adjustment for these covariates. Also, residual confounding bias in CPRD analysis cannot be ruled out since there was no information on genetic and lifestyle covariates, such as diet, education, and physical activity, which highlights the added value of MR analysis with genetic proxies.

In conclusion, our study provides strong support to the hypothesis that ADRD occurs less frequently in older adults previously diagnosed with cancer than those who remain free of cancer diagnosis. Our cohort analysis capitalized on the richness of the available longitudinal data in CPRD to better delineate the enigmatic associations between cancer and risk of dementia, whilst accounting for several methodological and statistical considerations. Evidence from Mendelian Randomization analyses suggests the presence of vertical pleiotropic causal links between overall cancer, breast and lung cancers and risk of ADRD. These findings allude to a potential neuro-protective effect of biological processes implicated in cancer or an unforeseen neuro-protective effect of cancer chemotherapies as plausible drivers for the observed associations. Further studies aimed at unravelling the crosstalk between cancer biology, brain ageing, and neurodegeneration could strategically inform the identification of novel and effective therapies for AD and related dementias.

## Supplementary file

Supplementary material, approximately 348 KB.

## Notes

*Ethical standards:* CPRD has NHS Health Research Authority (HRA) Research Ethics Committee (REC) approval to allow the collection and release of anonymised primary care data for observational research. Each year CPRD obtains Section 251 regulatory support through the HRA Confidentiality Advisory Group (CAG), to enable patient identifiers, without accompanying clinical data, to flow from CPRD contributing GP practices in England to NHS Digital, for the purposes of data linkage. The protocol for this study was approved by CPRD’s Independent Scientific Advisory Committee (ISAC) for Medicines and Healthcare products Regulatory Agency (MHRA) database research (protocol number: 16_219R2). Select general practices consent to this process at a practice level with individual patients having the right to opt-out.
