# Supplementary material for: Lower Incidence of Dementia Following Cancer Diagnoses: Evidence from a Large Cohort and Mendelian Randomization Study
Source: J Prev Alzheimers Dis. 2024 Jul 9;11(5):1397–405. doi: 10.14283/jpad.2024.135 (PMC11436397; doi:10.14283/jpad.2024.135)

**Supplementary file**

**Supplementary Table 1. CPRD codelist for overall cancer and specific types of cancer**

**Supplementary Table 2. CPRD codelist for dementia diagnosis and drug prescription**

**Supplementary Table 3. Sensitivity analyses of association between cancer and risk of dementia incidence**

**Supplementary Table 4. Sensitivity analyses of Mendelian randomization analysis on overall and subtypes of cancer and risk of Alzheimer’s disease**

**Supplementary Figure 1. Forest plots of Mendelian randomization analysis on overall and subtypes of cancer and risk of Alzheimer’s disease**

**Supplementary Figure 2. Funnel plots of Mendelian randomization analysis on overall and subtypes of cancer and risk of Alzheimer’s disease**

**Supplementary Table 1a. CPRD codelist for overall cancer**

| **Medcode** | **Description** |
| --- | --- |
| 40766 | [M] Peripheral T-cell lymphoma NOS |
| 37688 | [M]Acinar cell carcinoma |
| 72440 | [M]Acinar cell tumour |
| 6316 | [M]Acute leukaemia NOS |
| 37410 | [M]Acute lymphoid leukaemia |
| 49327 | [M]Acute megakaryoblastic leukaemia |
| 54585 | [M]Acute myeloid leukaemia |
| 46263 | [M]Acute myelomonocytic leukaemia |
| 57316 | [M]Acute promyelocytic leukaemia |
| 56794 | [M]Adenocarcinoid tumour |
| 73275 | [M]Adenocarcinoma in adenomatous polposis coli |
| 52326 | [M]Adenocarcinoma in adenomatous polyp |
| 73434 | [M]Adenocarcinoma in multiple adenomatous polyps |
| 44778 | [M]Adenocarcinoma in tubulovillous adenoma |
| 67342 | [M]Adenocarcinoma in villous adenoma |
| 8930 | [M]Adenocarcinoma NOS |
| 66000 | [M]Adenocarcinoma with apocrine metaplasia |
| 42553 | [M]Adenocarcinoma with cartilaginous and osseous metaplasia |
| 94810 | [M]Adenocarcinoma with spindle cell metaplasia |
| 16146 | [M]Adenocarcinoma with squamous metaplasia |
| 28272 | [M]Adenocarcinoma, intestinal type |
| 5455 | [M]Adenocarcinoma, metastatic, NOS |
| 2272 | [M]Adenocarcinomas |
| 33775 | [M]Adenoid cystic carcinoma |
| 35975 | [M]Adenoma or adenocarcinoma NOS |
| 19731 | [M]Adenoma or or adenocarcinoma in polyposis coli |
| 19091 | [M]Adenomas and adenocarcinomas |
| 18255 | [M]Adenomatous and adenocarcinomatous polyps |
| 41702 | [M]Adenomatous and adenocarcinomatous polyps of colon |
| 36286 | [M]Adenomatous or adenocarcinomatous polyp NOS |
| 39875 | [M]Adenomatous or adenocarcinomatous polyps of the colon NOS |
| 12580 | [M]Adenosquamous carcinoma |
| 60775 | [M]Adrenal cortical carcinoma |
| 40883 | [M]Adrenal cortical tumours |
| 62256 | [M]Adrenal cortical tumours NOS |
| 50519 | [M]Adrenal rest tumour |
| 29335 | [M]Adult T-cell leukaemia/lymphoma |
| 72310 | [M]Aleukaemic leukaemia NOS |
| 57802 | [M]Alveolar adenocarcinoma |
| 36530 | [M]Alveolar cell carcinoma |
| 67018 | [M]Aortic body tumour |
| 38575 | [M]Apocrine adenocarcinoma |
| 24312 | [M]Apocrine adenoma and adenocarcinomas |
| 104973 | [M]Apocrine adenoma or adenocarcinoma NOS |
| 38454 | [M]Basaloid carcinoma |
| 72277 | [M]Basophil carcinoma |
| 106197 | [M]Basophilic leukaemia |
| 40438 | [M]Bile duct carcinoma |
| 41313 | [M]Bile duct cystadenocarcinoma |
| 70516 | [M]Biliary tract adenomas and adenocarcinomas |
| 22071 | [M]Blast cell leukaemia |
| 64963 | [M]Blastic leukaemia |
| 62881 | [M]Blood vessel tumour NOS |
| 58976 | [M]Blood vessel tumours |
| 44793 | [M]Brenner tumour NOS |
| 71490 | [M]Brenner tumour, borderline malignancy |
| 70383 | [M]Brenner tumour, malignant |
| 25810 | [M]Brenner tumours |
| 16723 | [M]Bronchiolar carcinoma |
| 34015 | [M]Bronchiolo-alveolar adenocarcinoma |
| 50928 | [M]Burkitt's cell leukaemia |
| 47920 | [M]C cell carcinoma |
| 59274 | [M]Calcifying epithelial odontogenic tumour |
| 38444 | [M]Carcinoid tumour NOS |
| 111172 | [M]Carcinoid tumour, argentaffin, malignant |
| 39130 | [M]Carcinoid tumour, argentaffin, NOS |
| 34110 | [M]Carcinoid tumour, malignant |
| 100625 | [M]Carcinoid tumour, nonargentaffin, malignant |
| 99296 | [M]Carcinoid tumour, nonargentaffin, NOS |
| 3923 | [M]Carcinoid tumours |
| 45573 | [M]Carcinoid tumours NOS |
| 37510 | [M]Carcinoma in pleomorphic adenoma |
| 8695 | [M]Carcinoma NOS |
| 12609 | [M]Carcinoma, anaplastic type, NOS |
| 59240 | [M]Carcinoma, diffuse type |
| 3152 | [M]Carcinoma, metastatic, NOS |
| 21609 | [M]Carcinoma, undifferentiated type, NOS |
| 16692 | [M]Carcinomatosis |
| 36209 | [M]Carotid body tumour |
| 67913 | [M]Ceruminous adenoma and adenocarcinoma |
| 8711 | [M]Cholangiocarcinoma |
| 32442 | [M]Chondromatous giant cell tumour |
| 67712 | [M]Choriocarcinoma |
| 54627 | [M]Choriocarcinoma combined with teratoma |
| 68456 | [M]Chromophobe carcinoma |
| 31750 | [M]Chronic leukaemia NOS |
| 41500 | [M]Chronic lymphoid leukaemia |
| 52942 | [M]Chronic myeloid leukaemia |
| 48049 | [M]Chronic myelomonocytic leukaemia |
| 37354 | [M]Clear cell adenocarcinoma NOS |
| 72192 | [M]Clear cell adenoma or adenocarcinoma NOS |
| 36882 | [M]Clear cell adenomas and adenocarcinomas |
| 65216 | [M]Cloacogenic carcinoma |
| 30416 | [M]Colloid adenocarcinoma |
| 107299 | [M]Combined hepatocellular carcinoma and cholangiocarcinoma |
| 58131 | [M]Comedocarcinoma NOS |
| 62871 | [M]Comedocarcinoma, noninfiltrating |
| 50140 | [M]Cribriform carcinoma |
| 34879 | [M]Cylindroid adenocarcinoma |
| 34000 | [M]Cystadenocarcinoma NOS |
| 34984 | [M]Cystadenoma and carcinoma |
| 65207 | [M]Cystadenoma or carcinoma NOS |
| 21833 | [M]Duct carcinoma NOS |
| 28941 | [M]Embryonal carcinoma NOS |
| 37621 | [M]Endodermal sinus tumour |
| 28388 | [M]Endometrioid adenoma or carcinoma NOS |
| 8606 | [M]Endometrioid adenomas and carcinomas |
| 9447 | [M]Endometrioid carcinoma |
| 36876 | [M]Eosinophil carcinoma |
| 71377 | [M]Eosinophilic leukaemia |
| 107773 | [M]Eosinophilic leukaemia NOS |
| 57713 | [M]Eosinophilic leukaemias |
| 56600 | [M]Epidermoid carcinoma NOS |
| 57513 | [M]Epidermoid carcinoma, keratinising type |
| 38770 | [M]Epithelial-myoepithelial carcinoma |
| 70935 | [M]Erythroleukaemia |
| 100927 | [M]Erythroleukaemia NOS |
| 46444 | [M]Erythroleukaemias |
| 21741 | [M]Follicular adenocarcinoma NOS |
| 61467 | [M]Follicular adenocarcinoma, trabecular type |
| 59918 | [M]Follicular adenocarcinoma, well differentiated type |
| 21847 | [M]Follicular carcinoma |
| 45814 | [M]G cell tumour NOS |
| 26858 | [M]Gastrinoma and carcinomas |
| 43594 | [M]Gastrinoma or carcinoma NOS |
| 95008 | [M]Gelatinous adenocarcinoma |
| 48048 | [M]Giant cell and spindle cell carcinoma |
| 35474 | [M]Giant cell carcinoma |
| 37830 | [M]Giant cell tumour NOS |
| 38477 | [M]Giant cell tumour of bone NOS |
| 68956 | [M]Giant cell tumour of bone, malignant |
| 45364 | [M]Giant cell tumour of soft parts NOS |
| 22330 | [M]Giant cell tumours |
| 45953 | [M]Glomus jugulare tumour |
| 45969 | [M]Glomus tumour |
| 69210 | [M]Goblet cell tumour |
| 92275 | [M]Gonadal stromal tumour |
| 34096 | [M]Granular cell carcinoma |
| 18266 | [M]Granular cell tumour NOS |
| 41803 | [M]Granular cell tumours and alveolar soft part sarcoma |
| 37723 | [M]Granulocytic leukaemia NOS |
| 6751 | [M]Granulosa cell tumour NOS |
| 31609 | [M]Granulosa cell tumour, malignant |
| 48957 | [M]Granulosa cell-theca cell tumour |
| 52266 | [M]Grawitz tumour |
| 36646 | [M]Haemangiomatous tumours |
| 5915 | [M]Hairy cell leukaemia |
| 53987 | [M]Hepatobiliary adenoma or carcinoma NOS |
| 36031 | [M]Hepatobiliary tract adenomas and carcinomas |
| 40240 | [M]Hepatocellular carcinoma NOS |
| 46771 | [M]Hepatocellular carcinoma, fibrolamellar |
| 110147 | [M]Hepatocholangiocarcinoma |
| 39734 | [M]Hilar cell tumour |
| 29008 | [M]Hurthle cell adenocarcinoma |
| 27697 | [M]Hypernephroid tumour |
| 104147 | [M]Infantile embryonal carcinoma |
| 39760 | [M]Infiltrating duct and lobular carcinoma |
| 8351 | [M]Infiltrating duct carcinoma |
| 7319 | [M]Infiltrating ductular carcinoma |
| 32472 | [M]Inflammatory carcinoma |
| 99219 | [M]Interstitial cell tumour NOS |
| 3969 | [M]Intracystic carcinoma NOS |
| 27728 | [M]Intraductal carcinoma, noninfiltrating NOS |
| 30189 | [M]Intraductal papillary adenocarcinoma with invasion |
| 19041 | [M]Intraepidermal carcinoma NOS |
| 21914 | [M]Intraepithelial carcinoma NOS |
| 93175 | [M]Intraosseous carcinoma |
| 52178 | [M]Intravascular bronchial alveolar tumour |
| 63102 | [M]Islet cell carcinoma |
| 40359 | [M]Juvenile breast carcinoma |
| 52963 | [M]Juvenile granulosa cell tumour |
| 108964 | [M]Juvenile myelomonocytic leukaemia |
| 49900 | [M]Klatskin's tumour |
| 53694 | [M]Krukenberg tumour |
| 25961 | [M]Large cell carcinoma NOS |
| 42297 | [M]Leukaemia NOS |
| 41734 | [M]Leukaemia NOS |
| 59929 | [M]Leukaemia unspecified, NOS |
| 4637 | [M]Leukaemias |
| 40420 | [M]Leukaemias unspecified |
| 31170 | [M]Leydig cell tumour |
| 73623 | [M]Leydig cell tumour NOS |
| 95373 | [M]Leydig cell tumour, malignant |
| 59995 | [M]Lipid cell tumour of ovary |
| 25641 | [M]Liver cell carcinoma |
| 12427 | [M]Lobular carcinoma NOS |
| 40415 | [M]Lymphangiomatous tumours |
| 20635 | [M]Lymphatic leukaemia |
| 96713 | [M]Lymphatic vessel tumour NOS |
| 71481 | [M]Lymphatic vessel tumours |
| 34352 | [M]Lymphoblastic lymphoma NOS |
| 21463 | [M]Lymphocytic lymphoma NOS |
| 45510 | [M]Lymphoepithelial carcinoma |
| 12146 | [M]Lymphoid leukaemia NOS |
| 48155 | [M]Lymphoid leukaemias |
| 99797 | [M]Malignant giant cell tumour of soft parts |
| 36114 | [M]Malignant lymphoma NOS |
| 32213 | [M]Malignant tumour, fusiform cell type |
| 24511 | [M]Malignant tumour, giant cell type |
| 22156 | [M]Malignant tumour, small cell type |
| 92385 | [M]Mast cell tumour NOS |
| 37128 | [M]Mast cell tumours |
| 16677 | [M]Medullary carcinoma NOS |
| 50946 | [M]Medullary carcinoma with amyloid stroma |
| 98883 | [M]Medullary carcinoma with lymphoid stroma |
| 72222 | [M]Megakaryocytic leukaemia |
| 24551 | [M]Melanocarcinoma |
| 64874 | [M]Melanotic neuroectodermal tumour |
| 32641 | [M]Merkel cell carcinoma |
| 49811 | [M]Mesodermal mixed tumour |
| 54874 | [M]Metastatic signet ring cell carcinoma |
| 13574 | [M]Metatypical carcinoma |
| 66502 | [M]Miscellaneous bone tumour NOS |
| 9102 | [M]Miscellaneous bone tumours |
| 108316 | [M]Miscellaneous leukaemia NOS |
| 73066 | [M]Miscellaneous leukaemias |
| 28950 | [M]Miscellaneous tumour NOS |
| 64314 | [M]Miscellaneous tumours |
| 111238 | [M]Mixed cell adenoma or adenocarcinoma NOS |
| 35071 | [M]Mixed germ cell tumour |
| 98825 | [M]Mixed islet cell and exocrine adenocarcinoma |
| 44217 | [M]Mixed tumour NOS |
| 66607 | [M]Mixed tumour, malignant, NOS |
| 73088 | [M]Monocytic leukaemia NOS |
| 12497 | [M]Mucinous adenocarcinoma |
| 40632 | [M]Mucinous adenoma and adenocarcinoma |
| 64796 | [M]Mucinous adenoma or adenocarcinoma NOS |
| 51656 | [M]Mucinous cystadenocarcinoma NOS |
| 44074 | [M]Mucin-producing adenocarcinoma |
| 55468 | [M]Mucocarcinoid tumour, malignant |
| 28625 | [M]Mucoepidermoid carcinoma |
| 59100 | [M]Mucoepidermoid tumour |
| 40622 | [M]Mucoid cell carcinoma |
| 59284 | [M]Mucous adenocarcinoma |
| 21173 | [M]Mullerian mixed tumour |
| 71850 | [M]Myeloid leukaemia NOS |
| 35697 | [M]Myeloid leukaemias |
| 66694 | [M]Naegeli-type monocytic leukaemia |
| 67966 | [M]Naevocarcinoma |
| 18690 | [M]Nerve sheath tumour |
| 35284 | [M]Nerve sheath tumour NOS |
| 26253 | [M]Neuroendocrine carcinoma |
| 68757 | [M]Nonencapsulated sclerosing carcinoma |
| 102593 | [M]Noninfiltrating intraductal papillary adenocarcinoma |
| 106519 | [M]Non-small cell carcinoma |
| 9156 | [M]Oat cell carcinoma |
| 101568 | [M]Odontogenic tumour NOS |
| 55833 | [M]Odontogenic tumour NOS |
| 72443 | [M]Odontogenic tumour, malignant |
| 41274 | [M]Odontogenic tumours |
| 58902 | [M]Olfactory neurogenic tumour |
| 53129 | [M]Oncytic adenocarcinoma |
| 62330 | [M]Other myeloid leukaemia NOS |
| 17151 | [M]Ovarian cystadenoma or carcinoma |
| 18638 | [M]Ovarian mucinous tumour |
| 39007 | [M]Ovarian papillary tumour |
| 30541 | [M]Ovarian serous tumour |
| 21435 | [M]Ovarian stromal tumour |
| 71497 | [M]Oxyphilic adenocarcinoma |
| 73662 | [M]Oxyphilic adenoma or adenocarcinoma NOS |
| 62199 | [M]Oxyphilic adenomas and adenocarcinomas |
| 42542 | [M]Paget's disease and infiltrating breast duct carcinoma |
| 12480 | [M]Paget's disease and intraductal carcinoma of breast |
| 21659 | [M]Pancreatic adenoma or carcinoma NOS |
| 8032 | [M]Pancreatic adenomas and carcinomas |
| 35348 | [M]Papillary adenocarcinoma NOS |
| 96494 | [M]Papillary adenoma or adenocarcinoma NOS |
| 42273 | [M]Papillary adenomas and adenocarcinomas |
| 46761 | [M]Papillary and follicular adenocarcinoma |
| 10541 | [M]Papillary carcinoma NOS |
| 65051 | [M]Papillary cystadenocarcinoma, NOS |
| 72684 | [M]Papillary cystic tumour |
| 67912 | [M]Papillary epidermoid carcinoma |
| 54749 | [M]Papillary mucinous cystadenocarcinoma |
| 44930 | [M]Papillary serous cystadenocarcinoma |
| 9712 | [M]Papillary transitional cell carcinoma |
| 69421 | [M]Paraganglioma or glomus tumour NOS |
| 24924 | [M]Paragangliomas and glomus tumours |
| 42169 | [M]Parathyroid adenoma or adenocarcinoma NOS |
| 4217 | [M]Parathyroid adenomas and adenocarcinomas |
| 107884 | [M]Peripheral neuroectodermal tumour |
| 57422 | [M]Pituitary adenoma or carcinoma NOS |
| 26120 | [M]Pituitary adenomas and carcinomas |
| 110349 | [M]Plasma cell leukaemia NOS |
| 64618 | [M]Plasma cell leukaemias |
| 64068 | [M]Plasma cell tumour NOS |
| 99702 | [M]Plasma cell tumour, malignant |
| 43459 | [M]Plasma cell tumours |
| 26413 | [M]Pleomorphic carcinoma |
| 69300 | [M]Polygonal cell carcinoma |
| 43189 | [M]Precancerous melanosis NOS |
| 41695 | [M]Primitive neuroectodermal tumour |
| 46048 | [M]Prolymphocytic leukaemia |
| 66876 | [M]Pseudomucinous adenocarcinoma |
| 54276 | [M]Pseudosarcomatous carcinoma |
| 8101 | [M]Renal adenoma and carcinoma |
| 35467 | [M]Renal adenoma or carcinoma NOS |
| 10668 | [M]Renal cell carcinoma |
| 36221 | [M]Respiratory tract adenoma or adenocarcinoma NOS |
| 26848 | [M]Respiratory tract adenomas and adenocarcinomas |
| 73023 | [M]Retinal angle tumour |
| 66541 | [M]Round cell carcinoma |
| 100111 | [M]Schneiderian carcinoma |
| 48223 | [M]Scirrhous adenocarcinoma |
| 34269 | [M]Sebaceous adenocarcinoma |
| 28291 | [M]Sebaceous adenoma and adenocarcinoma |
| 91842 | [M]Sebaceous adenoma or adenocarcinoma NOS |
| 9366 | [M]Secondary carcinoma |
| 67701 | [M]Secretory breast carcinoma |
| 38442 | [M]Serous cystadenocarcinoma, NOS |
| 95150 | [M]Serous surface papillary carcinoma |
| 29580 | [M]Sertoli cell carcinoma |
| 38979 | [M]Sertoli cell tumour |
| 18065 | [M]Sertoli-Leydig cell tumour |
| 61115 | [M]Sex cord tumour with annular tubules |
| 54654 | [M]Sex cord-stromal tumour |
| 39038 | [M]Signet ring carcinoma |
| 94438 | [M]Signet ring carcinoma NOS |
| 61588 | [M]Signet ring cell carcinoma |
| 95046 | [M]Skin appendage adenoma and carcinoma |
| 68783 | [M]Skin appendage carcinoma |
| 9291 | [M]Small cell carcinoma NOS |
| 67970 | [M]Small cell carcinoma, fusiform cell type |
| 30988 | [M]Small cell carcinoma, intermediate cell |
| 21217 | [M]Small cell-large cell carcinoma |
| 37081 | [M]Smooth muscle tumour NOS |
| 55116 | [M]Soft tissue tumour or sarcoma NOS |
| 17366 | [M]Soft tissue tumours and sarcomas NOS |
| 94083 | [M]Solid carcinoma NOS |
| 112200 | [M]Solid carcinoma with amyloid stroma |
| 61984 | [M]Spheroidal cell carcinoma |
| 6966 | [M]Spindle cell carcinoma |
| 57680 | [M]Spinous cell carcinoma |
| 63570 | [M]Stem cell leukaemia |
| 72179 | [M]Subacute leukaemia NOS |
| 106483 | [M]Subacute myeloid leukaemia |
| 71895 | [M]Superficial spreading adenocarcinoma |
| 71627 | [M]Sweat gland adenocarcinoma |
| 52496 | [M]Sweat gland adenoma and adenocarcinomas |
| 67354 | [M]Sweat gland tumour NOS |
| 37542 | [M]Teratocarcinoma |
| 21319 | [M]Testicular stromal tumour |
| 69299 | [M]Thrombocytic leukaemia |
| 19263 | [M]Thyroid adenoma and adenocarcinoma |
| 38685 | [M]Thyroid adenoma or adenocarcinoma NOS |
| 98781 | [M]Trabecular adenocarcinoma |
| 6436 | [M]Transitional cell carcinoma NOS |
| 58798 | [M]Transitional cell carcinoma, spindle cell type |
| 33897 | [M]Transitional cell papilloma or carcinoma NOS |
| 1950 | [M]Transitional cell papillomas and carcinomas |
| 40492 | [M]Triton tumour, malignant |
| 60045 | [M]Tubular adenocarcinoma |
| 39148 | [M]Tubular adenoma or adenocarcinoma NOS |
| 6746 | [M]Tubular adenomas and adenocarcinomas |
| 8627 | [M]Tumour cells, malignant |
| 100112 | [M]Tumour embolism |
| 100590 | [M]Tumour embolus |
| 21207 | [M]Tumour morphology |
| 68679 | [M]Tumourlet |
| 50753 | [M]Turban tumour |
| 33508 | [M]Unspecified tumour cell NOS |
| 12388 | [M]Urothelial carcinoma |
| 34395 | [M]Verrucous carcinoma NOS |
| 43717 | [M]Verrucous epidermoid carcinoma |
| 27849 | [M]Villous adenocarcinoma |
| 50108 | [M]Villous adenoma or adenocarcinoma NOS |
| 6920 | [M]Villous adenomas and adenocarcinomas |
| 112235 | [M]Water-clear cell adenocarcinoma |
| 17314 | [M]Wilms' tumour |
| 20350 | [M]Yolk sac tumour |
| 30547 | [V]Follow-up exam after radiotherapy for malignant neoplasm |
| 31561 | [V]Follow-up examination aft surgery for malignant neoplasm |
| 53477 | [V]Follow-up examination after chemotherapy for leukaemia |
| 44421 | [V]Folow-up exam aft other treatment for malignant neoplasm |
| 36321 | [V]Folow-up exam aft unspec treatment for malignant neoplasm |
| 36693 | [V]Personal history of leukaemia |
| 94597 | [V]Personal history of lymphoid leukaemia |
| 64568 | [V]Personal history of malig neop of gastrointestinal tract |
| 62814 | [V]Personal history of malig neop of gastrointestinal tract |
| 49289 | [V]Personal history of malig neop of trachea/bronchus/lung |
| 72262 | [V]Personal history of malig neop other intrathoracic organ |
| 12106 | [V]Personal history of malignant neoplasm |
| 61655 | [V]Personal history of malignant neoplasm - accessory sinus |
| 68018 | [V]Personal history of malignant neoplasm of anus |
| 35816 | [V]Personal history of malignant neoplasm of bladder |
| 46282 | [V]Personal history of malignant neoplasm of bone |
| 48085 | [V]Personal history of malignant neoplasm of brain |
| 16639 | [V]Personal history of malignant neoplasm of breast |
| 32246 | [V]Personal history of malignant neoplasm of bronchus |
| 23936 | [V]Personal history of malignant neoplasm of cervix uteri |
| 103100 | [V]Personal history of malignant neoplasm of eye |
| 9444 | [V]Personal history of malignant neoplasm of genital organ |
| 109429 | [V]Personal history of malignant neoplasm of genital organ |
| 47683 | [V]Personal history of malignant neoplasm of kidney |
| 28881 | [V]Personal history of malignant neoplasm of kidney |
| 57727 | [V]Personal history of malignant neoplasm of large intestine |
| 43311 | [V]Personal history of malignant neoplasm of larynx |
| 58177 | [V]Personal history of malignant neoplasm of liver |
| 29284 | [V]Personal history of malignant neoplasm of lung |
| 39863 | [V]Personal history of malignant neoplasm of nose |
| 51001 | [V]Personal history of malignant neoplasm of oesophagus |
| 52141 | [V]Personal history of malignant neoplasm of ovary |
| 37306 | [V]Personal history of malignant neoplasm of prostate |
| 62785 | [V]Personal history of malignant neoplasm of rectum |
| 47669 | [V]Personal history of malignant neoplasm of skin |
| 49447 | [V]Personal history of malignant neoplasm of stomach |
| 48808 | [V]Personal history of malignant neoplasm of testis |
| 35771 | [V]Personal history of malignant neoplasm of thyroid |
| 99931 | [V]Personal history of malignant neoplasm of tongue |
| 45803 | [V]Personal history of malignant neoplasm of tongue |
| 30322 | [V]Personal history of malignant neoplasm of urinary organ |
| 46779 | [V]Personal history of malignant neoplasm of uterine body |
| 110058 | [V]Personal history of myeloid leukaemia |
| 66457 | [V]Personal history of other specified malignant neoplasm |
| 68612 | [V]Personal history of unspecified malignant neoplasm |
| 97091 | [X]2ndry malignant neoplasm/bladder+oth+unsp urinary organs |
| 68332 | [X]2ndry malignant neoplasm/oth+unspec parts/nervous system |
| 66163 | [X]2ndry+unspcf malignant neoplasm lymph nodes/multi regions |
| 112035 | [X]HIV disease resulting in other malignant neoplasms |
| 112036 | [X]HIV disease resulting in unspecified malignant neoplasm |
| 47633 | [X]Malig neopl, overlap lesion brain & other part of CNS |
| 40749 | [X]Malignant neoplasm of bone and articular cartilage |
| 12499 | [X]Malignant neoplasm of breast |
| 40595 | [X]Malignant neoplasm of bronchus or lung, unspecified |
| 35180 | [X]Malignant neoplasm of digestive organs |
| 64309 | [X]Malignant neoplasm of endocrine gland, unspecified |
| 35285 | [X]Malignant neoplasm of eye, brain and other parts of cent |
| 55588 | [X]Malignant neoplasm of female genital organ, unspecified |
| 40598 | [X]Malignant neoplasm of female genital organs |
| 35186 | [X]Malignant neoplasm of ill-defined, secondary and unspeci |
| 45766 | [X]Malignant neoplasm of intestinal tract, part unspecified |
| 58973 | [X]Malignant neoplasm of lip, oral cavity and pharynx |
| 45262 | [X]Malignant neoplasm of male genital organ, unspecified |
| 40671 | [X]Malignant neoplasm of male genital organs |
| 50292 | [X]Malignant neoplasm of mediastinum, part unspecified |
| 63925 | [X]Malignant neoplasm of meninges, unspecified |
| 40592 | [X]Malignant neoplasm of mesothelial and soft tissue |
| 39027 | [X]Malignant neoplasm of other specified sites |
| 95671 | [X]Malignant neoplasm of peritoneum, unspecified |
| 35325 | [X]Malignant neoplasm of respiratory and intrathoracic orga |
| 56121 | [X]Malignant neoplasm of skin, unspecified |
| 40608 | [X]Malignant neoplasm of thyroid and other endocrine glands |
| 45260 | [X]Malignant neoplasm of urinary organ, unspecified |
| 35113 | [X]Malignant neoplasm of urinary tract |
| 64497 | [X]Malignant neoplasm of uterine adnexa, unspecified |
| 60162 | [X]Malignant neoplasm overlapping lesion of skin |
| 52029 | [X]Malignant neoplasm without specification of site |
| 43151 | [X]Malignant neoplasm/bone+articular cartilage, unspecified |
| 73296 | [X]Malignant neoplasm/bones+articular cartilage/limb,unspfd |
| 41515 | [X]Malignant neoplasm/central nervous system, unspecified |
| 91457 | [X]Malignant neoplasm/connective + soft tissue,unspecified |
| 86997 | [X]Malignant neoplasm/ill-defined sites within resp system |
| 68027 | [X]Malignant neoplasm/other and unspecified cranial nerves |
| 57756 | [X]Malignant neoplasm/other specified female genital organs |
| 57191 | [X]Malignant neoplasm/other specified male genital organs |
| 63300 | [X]Malignant neoplasm/overlap lesion/bone+articulr cartilage |
| 66444 | [X]Malignant neoplasm/overlap lesion/heart,mediastinm+pleura |
| 96226 | [X]Malignant neoplasm/overlap lesion/other+ill-defined sites |
| 101668 | [X]Malignant neoplasm/peripheral nerves of trunk,unspecified |
| 111904 | [X]Malignant neoplasm/upper resp tract, part unspecified |
| 40740 | [X]Malignant neoplasms of lymphoid, haematopoietic and rela |
| 63598 | [X]Malignant neoplasms/independent (primary) multiple sites |
| 64897 | [X]Malignant neoplasms/independent(primary)multiple sites |
| 19144 | [X]Melanoma and other malignant neoplasms of skin |
| 7940 | [X]Non-Hodgkin's lymphoma NOS |
| 57184 | [X]Oth malignant neoplasm/skin of oth+unspecfd parts of face |
| 65165 | [X]Other leukaemia of unspecified cell type |
| 67029 | [X]Other lymphoid leukaemia |
| 89762 | [X]Other monocytic leukaemia |
| 61693 | [X]Other myeloid leukaemia |
| 43490 | [X]Other specified carcinomas of liver |
| 89329 | [X]Other specified leukaemias |
| 54253 | [X]Secondary malignant neoplasm of other specified sites |
| 57481 | [X]Secondary malignant neoplasm/oth+unspc respiratory organs |
| 88022 | [X]Secondary malignant neoplasm/oth+unspcfd digestive organs |
| 42539 | Acute erythraemia and erythroleukaemia |
| 110838 | Acute erythroid leukaemia |
| 4072 | Acute leukaemia NOS |
| 4251 | Acute lymphoid leukaemia |
| 108424 | Acute monoblastic leukaemia |
| 19974 | Acute monocytic leukaemia |
| 104788 | Acute myeloblastic leukaemia |
| 4413 | Acute myeloid leukaemia |
| 61500 | Acute myelomonocytic leukaemia |
| 27664 | Acute promyelocytic leukaemia |
| 26197 | Administration of cancer treatment |
| 37461 | Adult T-cell leukaemia |
| 104939 | Adult T-cell lymphoma/leukaemia (HTLV-1-associated) |
| 48145 | Anaemia in ovarian carcinoma |
| 9491 | Anal carcinoma |
| 107236 | Atypical chronic myeloid leukaemia, BCR/ABL negative |
| 104325 | B-cell acute lymphoblastic leukaemia |
| 104328 | B-cell chronic lymphocytic leukaemia |
| 108656 | B-cell prolymphocytic leukaemia |
| 65172 | Bone marrow: tumour cells |
| 62951 | Bone tumour/metast.irradiat. |
| 71304 | Burkitt's lymphoma NOS |
| 97577 | Burkitt's lymphoma of intra-abdominal lymph nodes |
| 100006 | Burkitt's lymphoma of intrathoracic lymph nodes |
| 59115 | Burkitt's lymphoma of lymph nodes of head, face and neck |
| 92380 | Burkitt's lymphoma of lymph nodes of inguinal region and leg |
| 5019 | Cancer chemotherapy |
| 10335 | Cancer confirmed |
| 10292 | Cancer diagnosis discussed |
| 22382 | Cancer diagnosis discussed with patient |
| 11075 | Cancer diagnosis discussed with significant other |
| 108081 | Cancer hormonal treatment drugs Band 1 |
| 54336 | Cancer hospital treatment completed |
| 11628 | Cancer of bowel |
| 1986 | Cancer of ovary |
| 59054 | Cancer rehabilitation and readaption |
| 32411 | Cancer treatment started |
| 2755 | Cancers |
| 36495 | Carcinoma common bile duct |
| 31393 | Carcinoma gallbladder |
| 9902 | Carcinoma of bone, connective tissue, skin and breast |
| 22163 | Carcinoma of caecum |
| 3357 | Carcinoma of digestive organs and peritoneum |
| 16874 | Carcinoma of genitourinary organ |
| 9984 | Carcinoma of lip |
| 24374 | Carcinoma of lip, oral cavity and pharynx |
| 8693 | Carcinoma of other and unspecified sites |
| 7219 | Carcinoma of rectum |
| 45307 | Carcinoma of respiratory tract and intrathoracic organs |
| 6170 | Carcinomatosis |
| 10851 | Cerebral tumour - malignant |
| 3230 | Cervical carcinoma (uterus) |
| 28003 | Choriocarcinoma |
| 5136 | Choriocarcinoma |
| 100786 | Chronic eosinophilic leukaemia |
| 31701 | Chronic granulocytic leukaemia |
| 16416 | Chronic leukaemia NOS |
| 27790 | Chronic lymphatic leukaemia |
| 107017 | Chronic lymphocytic leukaemia of B-cell type |
| 8625 | Chronic lymphoid leukaemia |
| 27458 | Chronic monocytic leukaemia |
| 10726 | Chronic myeloid leukaemia |
| 27520 | Chronic myeloid leukaemia NOS |
| 105957 | Chronic myeloid leukaemia, BCR/ABL positive |
| 22050 | Chronic myelomonocytic leukaemia |
| 102783 | Chronic neutrophilic leukaemia |
| 107052 | Clinical stage A chronic lymphocytic leukaemia |
| 106924 | Clinical stage B chronic lymphocytic leukaemia |
| 107163 | Clinical stage C chronic lymphocytic leukaemia |
| 9118 | Colonic cancer |
| 107456 | Crv smr - hi grade dyskaryosis? invasive squamous carcinoma |
| 92427 | Curettage of tumour of bone and graft HFQ |
| 91514 | Curettage of tumour of bone NEC |
| 44952 | Date cancer diagnosis received in primary care |
| 11590 | Debulking of tumour of unspecified organ |
| 97775 | Diencephalic syndrome secondary to tumour |
| 65180 | Diffuse non-Hodgkin's lymphoma undifferentiated (diffuse) |
| 55675 | Endocrine tumour of pancreas |
| 55354 | Endoscopic destruction of bladder tumour by laser |
| 106056 | Epithelioid trophoblastic tumour |
| 18270 | Excision malignant skin tumour |
| 97201 | Excision of tumour of bone |
| 87871 | Excision of tumour of bone NEC |
| 105889 | Follicular lymphoma grade 1 |
| 105095 | Follicular lymphoma grade 2 |
| 107166 | Follicular lymphoma grade 3 |
| 105020 | Follicular lymphoma grade 3a |
| 107973 | Follicular lymphoma grade 3b |
| 17182 | Follicular lymphoma NOS |
| 100210 | Gastrointestinal stromal tumour |
| 10178 | Gleason grading of prostate cancer |
| 15169 | Glomus tumour |
| 17177 | H/O: * leukaemia |
| 7628 | H/O: cancer |
| 7900 | H/O: carcinoma |
| 17064 | H/O: malignant neoplasm (*) |
| 102314 | H/O: prostate cancer |
| 87335 | Hairy cell leukaemia |
| 102686 | Has cancer key worker |
| 22187 | Hepatocellular carcinoma |
| 101700 | Hereditary nonpolyposis colon cancer |
| 108715 | Histiocytic leukaemia |
| 29789 | Histiocytic tumour NOS |
| 105324 | HIV disease resulting in multiple malignant neoplasms |
| 67575 | HIV disease resulting in unspecified malignant neoplasm |
| 106349 | Hodgkin lymphoma NOS |
| 101836 | Human immunodeficiency virus with secondary cancers |
| 105069 | Juvenile myelomonocytic leukaemia |
| 4250 | Leukaemia NOS |
| 25191 | Leukaemia of unspecified cell type |
| 105488 | Local recurrence of malignant tumour of breast |
| 105388 | Local recurrence of malignant tumour of urinary bladder |
| 2587 | Lung cancer |
| 4222 | Lymphatic leukaemia |
| 19372 | Lymphoid leukaemia |
| 38914 | Lymphoid leukaemia NOS |
| 72197 | Lymphosarcoma cell leukaemia |
| 24456 | Malig neop auditory tube, middle ear and mastoid air cells |
| 73537 | Malig neop auditory tube, middle ear, mastoid air cells NOS |
| 73718 | Malig neop connective and soft tissue head, face, neck NOS |
| 90546 | Malig neop connective and soft tissue hip and leg NOS |
| 54965 | Malig neop connective and soft tissue of popliteal space |
| 65233 | Malig neop connective and soft tissue other specified site |
| 53989 | Malig neop connective and soft tissue upper limb/shoulder |
| 104913 | Malig neop connective soft tissue upper limb/shoulder NOS |
| 98813 | Malig neop eyeball excl conjunctiva, cornea, retina, choroid |
| 26652 | Malig neop nasal cavities, middle ear and accessory sinuses |
| 18608 | Malig neop of bone, connective tissue, skin and breast |
| 41011 | Malig neop of bone, connective tissue, skin and breast NOS |
| 19389 | Malig neop of bone, connective tissue, skin and breast OS |
| 43475 | Malig neop of connective and soft tissue head, face and neck |
| 60247 | Malig neop of connective and soft tissue of abdomen NOS |
| 66488 | Malig neop of connective and soft tissue of abdominal wall |
| 66088 | Malig neop of connective and soft tissue of hip and leg |
| 67324 | Malig neop of connective and soft tissue of inguinal region |
| 30542 | Malig neop of connective and soft tissue of lower leg |
| 58836 | Malig neop of connective and soft tissue of pelvis NOS |
| 98408 | Malig neop of connective and soft tissue of thorax NOS |
| 44805 | Malig neop of connective and soft tissue thigh and upper leg |
| 57471 | Malig neop of connective and soft tissue trunk unspecified |
| 64195 | Malig neop of endocrine gland or related structure NOS |
| 13559 | Malig neop of kidney and other unspecified urinary organs |
| 4555 | Malig neop of other and unspecified female genital organs |
| 65458 | Malig neop of other and unspecified parts of nervous system |
| 30511 | Malig neop of other endocrine glands and related structures |
| 100232 | Malig neop of other site of heart, thymus and mediastinum |
| 34075 | Malig neop of respiratory tract and intrathoracic organs |
| 65880 | Malig neop of scapula and long bones of upper arm NOS |
| 65793 | Malig neop of upper respiratory tract, part unspecified |
| 11009 | Malig neop oth/ill-defined sites digestive tract/peritoneum |
| 96971 | Malig neop other site nasal cavity, middle ear and sinuses |
| 55659 | Malig neop other site rectum, rectosigmoid junction and anus |
| 46114 | Malig neop other/ill-defined sites lip, oral cavity, pharynx |
| 44356 | Malig neop other/ill-defined sites resp/intrathoracic organs |
| 59718 | Malig neop pituitary gland or craniopharyngeal duct NOS |
| 62399 | Malig neop skin of ear and external auricular canal NOS |
| 110192 | Malig neopl of connective and soft tissue - sacrum or coccyx |
| 24235 | Malig neopl peripheral nerves and autonomic nervous system |
| 53504 | Malig neopl, overlap lesion brain & other part of CNS |
| 94272 | Malig neoplasm of connective and soft tissues of lumb spine |
| 104139 | Malig neoplasm of connective and soft tissues of thor spine |
| 15027 | Malignant lymphoma NOS |
| 12335 | Malignant lymphoma NOS |
| 42579 | Malignant lymphoma NOS of intra-abdominal lymph nodes |
| 71262 | Malignant lymphoma NOS of intrapelvic lymph nodes |
| 72725 | Malignant lymphoma NOS of intrathoracic lymph nodes |
| 63105 | Malignant lymphoma NOS of lymph node inguinal region and leg |
| 34089 | Malignant lymphoma NOS of lymph nodes of axilla and arm |
| 50696 | Malignant lymphoma NOS of lymph nodes of head, face and neck |
| 15504 | Malignant lymphoma NOS of lymph nodes of multiple sites |
| 60092 | Malignant lymphoma NOS of spleen |
| 57427 | Malignant lymphoma NOS of unspecified site |
| 17887 | Malignant lymphoma otherwise specified |
| 89657 | Malignant mast cell tumour NOS |
| 15036 | Malignant mast cell tumours |
| 57248 | Malignant neoplasm aryepiglottic fold, hypopharyngeal aspect |
| 15711 | Malignant neoplasm cerebrum (excluding lobes and ventricles) |
| 54103 | Malignant neoplasm gallbladder and extrahepatic bile ducts |
| 15907 | Malignant neoplasm gallbladder/extrahepatic bile ducts NOS |
| 49301 | Malignant neoplasm lymphatic or haematopoietic tissue NOS |
| 30646 | Malignant neoplasm lymphatic or haematopoietic tissue OS |
| 15976 | Malignant neoplasm of abdomen |
| 63470 | Malignant neoplasm of abdominal oesophagus |
| 55246 | Malignant neoplasm of accessory sinus NOS |
| 65599 | Malignant neoplasm of acoustic nerve |
| 105797 | Malignant neoplasm of acromion |
| 33388 | Malignant neoplasm of adenoid |
| 61390 | Malignant neoplasm of adrenal cortex |
| 28148 | Malignant neoplasm of adrenal gland |
| 70824 | Malignant neoplasm of adrenal gland NOS |
| 94220 | Malignant neoplasm of adrenal medulla |
| 10949 | Malignant neoplasm of ampulla of Vater |
| 24370 | Malignant neoplasm of anal canal |
| 58121 | Malignant neoplasm of anterior 2/3 of tongue unspecified |
| 102142 | Malignant neoplasm of anterior 2/3 of tongue ventral surface |
| 46728 | Malignant neoplasm of anterior epiglottis |
| 73439 | Malignant neoplasm of anterior epiglottis NOS |
| 27715 | Malignant neoplasm of anterior mediastinum |
| 45408 | Malignant neoplasm of anterior portion of floor of mouth |
| 44139 | Malignant neoplasm of anterior wall of nasopharynx |
| 100918 | Malignant neoplasm of anterior wall of nasopharynx NOS |
| 65312 | Malignant neoplasm of anterior wall of stomach NEC |
| 19162 | Malignant neoplasm of anterior wall of urinary bladder |
| 27897 | Malignant neoplasm of anus unspecified |
| 47840 | Malignant neoplasm of aortic body |
| 50035 | Malignant neoplasm of aortic body and other paraganglia |
| 103995 | Malignant neoplasm of aortic body or paraganglia NOS |
| 18632 | Malignant neoplasm of appendix |
| 64686 | Malignant neoplasm of areola of female breast |
| 67884 | Malignant neoplasm of areola of male breast |
| 63460 | Malignant neoplasm of arytenoid cartilage |
| 10946 | Malignant neoplasm of ascending colon |
| 107916 | Malignant neoplasm of auditory (Eustachian) tube |
| 37618 | Malignant neoplasm of axilla NOS |
| 20685 | Malignant neoplasm of axillary tail of female breast |
| 68787 | Malignant neoplasm of back NOS |
| 48073 | Malignant neoplasm of basal ganglia |
| 43431 | Malignant neoplasm of base of tongue |
| 34409 | Malignant neoplasm of base of tongue dorsal surface |
| 41571 | Malignant neoplasm of bladder neck |
| 40810 | Malignant neoplasm of body of pancreas |
| 48743 | Malignant neoplasm of body of penis |
| 43572 | Malignant neoplasm of body of stomach |
| 7046 | Malignant neoplasm of body of uterus |
| 33617 | Malignant neoplasm of body of uterus NOS |
| 18314 | Malignant neoplasm of bone and articular cartilage |
| 16075 | Malignant neoplasm of bone and articular cartilage NOS |
| 59036 | Malignant neoplasm of bones of skull and face |
| 69146 | Malignant neoplasm of bones of skull and face NOS |
| 18617 | Malignant neoplasm of brain |
| 41520 | Malignant neoplasm of brain NOS |
| 44089 | Malignant neoplasm of brain stem |
| 68641 | Malignant neoplasm of brain stem NOS |
| 101778 | Malignant neoplasm of broad ligament |
| 3903 | Malignant neoplasm of bronchus or lung NOS |
| 30402 | Malignant neoplasm of buccal mucosa |
| 3811 | Malignant neoplasm of caecum |
| 72212 | Malignant neoplasm of calcaneum |
| 36731 | Malignant neoplasm of canthus |
| 32022 | Malignant neoplasm of cardia of stomach |
| 37859 | Malignant neoplasm of cardia of stomach NOS |
| 100584 | Malignant neoplasm of cardiac orifice of stomach |
| 22894 | Malignant neoplasm of cardio-oesophageal junction of stomach |
| 17391 | Malignant neoplasm of carina of bronchus |
| 57047 | Malignant neoplasm of carotid body |
| 69104 | Malignant neoplasm of carpal bone - lunate |
| 57988 | Malignant neoplasm of carpal bone - scaphoid |
| 106069 | Malignant neoplasm of carpal bones |
| 60035 | Malignant neoplasm of cartilage of ear |
| 71204 | Malignant neoplasm of cartilage of nose |
| 9622 | Malignant neoplasm of cauda equina |
| 31546 | Malignant neoplasm of central part of female breast |
| 45154 | Malignant neoplasm of cerebellum |
| 61399 | Malignant neoplasm of cerebral cortex |
| 110766 | Malignant neoplasm of cerebral dura mater |
| 28919 | Malignant neoplasm of cerebral meninges |
| 70104 | Malignant neoplasm of cerebral meninges NOS |
| 64557 | Malignant neoplasm of cerebral peduncle |
| 109473 | Malignant neoplasm of cerebral pia mater |
| 52511 | Malignant neoplasm of cerebral ventricles |
| 54133 | Malignant neoplasm of cerebrum NOS |
| 61695 | Malignant neoplasm of cervical oesophagus |
| 95505 | Malignant neoplasm of cervical stump |
| 46939 | Malignant neoplasm of cervical vertebra |
| 2747 | Malignant neoplasm of cervix uteri |
| 28311 | Malignant neoplasm of cervix uteri NOS |
| 31364 | Malignant neoplasm of cheek mucosa |
| 41931 | Malignant neoplasm of cheek NOS |
| 23861 | Malignant neoplasm of chest wall NOS |
| 15991 | Malignant neoplasm of choroid |
| 46789 | Malignant neoplasm of choroid plexus |
| 59041 | Malignant neoplasm of ciliary body |
| 66639 | Malignant neoplasm of clavicle |
| 53910 | Malignant neoplasm of clitoris |
| 46159 | Malignant neoplasm of cloacogenic zone |
| 46905 | Malignant neoplasm of coccygeal body |
| 66908 | Malignant neoplasm of coccygeal vertebra |
| 1220 | Malignant neoplasm of colon |
| 28163 | Malignant neoplasm of colon NOS |
| 96783 | Malignant neoplasm of commissure of lip |
| 7982 | Malignant neoplasm of common bile duct |
| 63657 | Malignant neoplasm of conjunctiva |
| 34451 | Malignant neoplasm of connective and other soft tissue |
| 45071 | Malignant neoplasm of connective and soft tissue of abdomen |
| 29160 | Malignant neoplasm of connective and soft tissue of axilla |
| 70463 | Malignant neoplasm of connective and soft tissue of buttock |
| 91586 | Malignant neoplasm of connective and soft tissue of finger |
| 54222 | Malignant neoplasm of connective and soft tissue of foot |
| 57482 | Malignant neoplasm of connective and soft tissue of fore-arm |
| 19321 | Malignant neoplasm of connective and soft tissue of hand |
| 102949 | Malignant neoplasm of connective and soft tissue of hip |
| 51965 | Malignant neoplasm of connective and soft tissue of pelvis |
| 59152 | Malignant neoplasm of connective and soft tissue of perineum |
| 50222 | Malignant neoplasm of connective and soft tissue of shoulder |
| 22290 | Malignant neoplasm of connective and soft tissue of thorax |
| 63988 | Malignant neoplasm of connective and soft tissue of thumb |
| 99572 | Malignant neoplasm of connective and soft tissue of toe |
| 15182 | Malignant neoplasm of connective and soft tissue, site NOS |
| 64345 | Malignant neoplasm of connective and soft tissue, upper arm |
| 86996 | Malignant neoplasm of connective tissue of orbit |
| 73992 | Malignant neoplasm of cornea |
| 72723 | Malignant neoplasm of cornu of corpus uteri |
| 59170 | Malignant neoplasm of corpus callosum |
| 45490 | Malignant neoplasm of corpus uteri NOS |
| 3213 | Malignant neoplasm of corpus uteri, excluding isthmus |
| 60403 | Malignant neoplasm of costal cartilage |
| 67763 | Malignant neoplasm of costo-vertebral joint |
| 99621 | Malignant neoplasm of cranial nerves |
| 101086 | Malignant neoplasm of cranial nerves NOS |
| 39899 | Malignant neoplasm of craniopharyngeal duct |
| 37805 | Malignant neoplasm of cricoid cartilage |
| 106569 | Malignant neoplasm of crystalline lens |
| 107878 | Malignant neoplasm of cuneiform cartilage |
| 72445 | Malignant neoplasm of cystic duct |
| 19475 | Malignant neoplasm of descended testis |
| 91509 | Malignant neoplasm of descended testis NOS |
| 10864 | Malignant neoplasm of descending colon |
| 54186 | Malignant neoplasm of diaphragm |
| 15709 | Malignant neoplasm of digestive organs and peritoneum |
| 51255 | Malignant neoplasm of digestive tract and peritoneum NOS |
| 44996 | Malignant neoplasm of dome of urinary bladder |
| 43642 | Malignant neoplasm of dorsal surface of tongue |
| 43781 | Malignant neoplasm of dorsum of tongue NOS |
| 18613 | Malignant neoplasm of duodenum |
| 96635 | Malignant neoplasm of ectopic pancreatic tissue |
| 95057 | Malignant neoplasm of ectopic site of female breast |
| 95323 | Malignant neoplasm of ectopic site of male breast |
| 63430 | Malignant neoplasm of endocardium |
| 57235 | Malignant neoplasm of endocervical canal |
| 53103 | Malignant neoplasm of endocervical gland |
| 48820 | Malignant neoplasm of endocervix |
| 50285 | Malignant neoplasm of endocervix NOS |
| 49400 | Malignant neoplasm of endometrium |
| 2890 | Malignant neoplasm of endometrium of corpus uteri |
| 72127 | Malignant neoplasm of epididymis |
| 55374 | Malignant neoplasm of epiglottis NOS |
| 26134 | Malignant neoplasm of epiglottis, free border |
| 53594 | Malignant neoplasm of ethmoid bone |
| 54636 | Malignant neoplasm of ethmoid sinus |
| 50297 | Malignant neoplasm of exocervix |
| 23433 | Malignant neoplasm of extrahepatic bile ducts |
| 74896 | Malignant neoplasm of extrahepatic bile ducts NOS |
| 20160 | Malignant neoplasm of eye |
| 54956 | Malignant neoplasm of eye NOS |
| 56718 | Malignant neoplasm of eyeball NOS |
| 43087 | Malignant neoplasm of eyelid including canthus |
| 49828 | Malignant neoplasm of fallopian tube |
| 51926 | Malignant neoplasm of faucial pillar |
| 26448 | Malignant neoplasm of faucial tonsil |
| 3968 | Malignant neoplasm of female breast |
| 9470 | Malignant neoplasm of female breast NOS |
| 20166 | Malignant neoplasm of female genital organ NOS |
| 56513 | Malignant neoplasm of femur |
| 50402 | Malignant neoplasm of fibula |
| 94427 | Malignant neoplasm of fifth metacarpal bone |
| 110993 | Malignant neoplasm of first metacarpal bone |
| 69927 | Malignant neoplasm of first metatarsal bone |
| 91035 | Malignant neoplasm of fixed part of tongue NOS |
| 94355 | Malignant neoplasm of flank NOS |
| 20092 | Malignant neoplasm of floor of mouth |
| 36716 | Malignant neoplasm of floor of mouth NOS |
| 92382 | Malignant neoplasm of fourth metatarsal bone |
| 63979 | Malignant neoplasm of frenulum linguae |
| 53599 | Malignant neoplasm of frontal bone |
| 42426 | Malignant neoplasm of frontal lobe |
| 15684 | Malignant neoplasm of frontal sinus |
| 68155 | Malignant neoplasm of fundus of corpus uteri |
| 32362 | Malignant neoplasm of fundus of stomach |
| 16105 | Malignant neoplasm of gallbladder |
| 94278 | Malignant neoplasm of gastro-oesophageal junction |
| 13252 | Malignant neoplasm of genitourinary organ |
| 52594 | Malignant neoplasm of genitourinary organ NOS |
| 38931 | Malignant neoplasm of genitourinary organ OS |
| 17841 | Malignant neoplasm of glans penis |
| 99913 | Malignant neoplasm of globus pallidus |
| 51795 | Malignant neoplasm of glomus jugulare |
| 91895 | Malignant neoplasm of glossoepiglottic fold |
| 99185 | Malignant neoplasm of glossopalatine fold |
| 318 | Malignant neoplasm of glottis |
| 72522 | Malignant neoplasm of great vessels |
| 55434 | Malignant neoplasm of greater curve of stomach unspecified |
| 47899 | Malignant neoplasm of greater vestibular (Bartholin's) gland |
| 43400 | Malignant neoplasm of gum |
| 93218 | Malignant neoplasm of gum NOS |
| 73530 | Malignant neoplasm of hand bones |
| 73556 | Malignant neoplasm of hand bones NOS |
| 37590 | Malignant neoplasm of hard palate |
| 55098 | Malignant neoplasm of head NOS |
| 8771 | Malignant neoplasm of head of pancreas |
| 68236 | Malignant neoplasm of head, neck and face |
| 58903 | Malignant neoplasm of head, neck and face NOS |
| 95644 | Malignant neoplasm of heart |
| 50289 | Malignant neoplasm of heart NOS |
| 66750 | Malignant neoplasm of heart, thymus and mediastinum NOS |
| 52537 | Malignant neoplasm of hepatic duct |
| 9088 | Malignant neoplasm of hepatic flexure of colon |
| 33444 | Malignant neoplasm of hilus of lung |
| 67236 | Malignant neoplasm of hippocampus |
| 37112 | Malignant neoplasm of histiocytic tissue |
| 61741 | Malignant neoplasm of humerus |
| 34012 | Malignant neoplasm of hypopharynx |
| 28451 | Malignant neoplasm of hypopharynx NOS |
| 70942 | Malignant neoplasm of hypothalamus |
| 33871 | Malignant neoplasm of ileum |
| 44609 | Malignant neoplasm of ilium |
| 57854 | Malignant neoplasm of inguinal region NOS |
| 65124 | Malignant neoplasm of interlobular bile ducts |
| 110775 | Malignant neoplasm of interlobular biliary canals |
| 17559 | Malignant neoplasm of intestinal tract, part unspecified |
| 16915 | Malignant neoplasm of intrahepatic bile ducts |
| 61643 | Malignant neoplasm of intrahepatic bile ducts NOS |
| 89593 | Malignant neoplasm of intrahepatic biliary passages |
| 58088 | Malignant neoplasm of intrahepatic gall duct |
| 97547 | Malignant neoplasm of intrathoracic site NOS |
| 59381 | Malignant neoplasm of iris |
| 59223 | Malignant neoplasm of ischium |
| 35795 | Malignant neoplasm of Islets of Langerhans |
| 43940 | Malignant neoplasm of isthmus of uterine body |
| 70729 | Malignant neoplasm of isthmus of uterine body NOS |
| 51818 | Malignant neoplasm of jaw NOS |
| 43479 | Malignant neoplasm of jejunum |
| 96003 | Malignant neoplasm of junction of hard and soft palate |
| 48519 | Malignant neoplasm of junctional region of epiglottis |
| 29462 | Malignant neoplasm of kidney or urinary organs NOS |
| 1599 | Malignant neoplasm of kidney parenchyma |
| 43761 | Malignant neoplasm of labia majora |
| 59362 | Malignant neoplasm of labia majora NOS |
| 58061 | Malignant neoplasm of labia minora |
| 71584 | Malignant neoplasm of lacrimal duct |
| 64817 | Malignant neoplasm of lacrimal gland |
| 101805 | Malignant neoplasm of lacrimal sac |
| 43111 | Malignant neoplasm of laryngeal cartilage |
| 97332 | Malignant neoplasm of laryngeal cartilage NOS |
| 39084 | Malignant neoplasm of laryngopharynx |
| 319 | Malignant neoplasm of larynx |
| 9237 | Malignant neoplasm of larynx NOS |
| 26813 | Malignant neoplasm of larynx, other specified site |
| 45986 | Malignant neoplasm of lateral portion of floor of mouth |
| 59004 | Malignant neoplasm of lateral wall of nasopharynx |
| 102205 | Malignant neoplasm of lateral wall of nasopharynx NOS |
| 56355 | Malignant neoplasm of lateral wall of oropharynx |
| 35963 | Malignant neoplasm of lateral wall of urinary bladder |
| 42193 | Malignant neoplasm of lesser curve of stomach unspecified |
| 24852 | Malignant neoplasm of lingual tonsil |
| 14712 | Malignant neoplasm of lip |
| 73614 | Malignant neoplasm of lip unspecified, buccal aspect |
| 61692 | Malignant neoplasm of lip unspecified, inner aspect |
| 68399 | Malignant neoplasm of lip unspecified, mucosa |
| 100144 | Malignant neoplasm of lip, oral aspect |
| 19415 | Malignant neoplasm of lip, oral cavity and pharynx |
| 39430 | Malignant neoplasm of lip, oral cavity and pharynx NOS |
| 37553 | Malignant neoplasm of lip, unspecified |
| 100906 | Malignant neoplasm of lip, unspecified, external |
| 94251 | Malignant neoplasm of lip, unspecified, lipstick area |
| 69761 | Malignant neoplasm of lip, vermilion border NOS |
| 8918 | Malignant neoplasm of liver and intrahepatic bile ducts |
| 38978 | Malignant neoplasm of liver and intrahepatic bile ducts NOS |
| 26393 | Malignant neoplasm of liver unspecified |
| 68055 | Malignant neoplasm of long bones of leg |
| 62630 | Malignant neoplasm of long bones of leg NOS |
| 97530 | Malignant neoplasm of lower buccal sulcus |
| 41958 | Malignant neoplasm of lower eyelid |
| 49360 | Malignant neoplasm of lower gum |
| 31399 | Malignant neoplasm of lower limb NOS |
| 67504 | Malignant neoplasm of lower lip, buccal aspect |
| 66384 | Malignant neoplasm of lower lip, external |
| 91843 | Malignant neoplasm of lower lip, frenulum |
| 71147 | Malignant neoplasm of lower lip, inner aspect |
| 96782 | Malignant neoplasm of lower lip, inner aspect NOS |
| 95480 | Malignant neoplasm of lower lip, lipstick area |
| 89909 | Malignant neoplasm of lower lip, mucosa |
| 94441 | Malignant neoplasm of lower lip, oral aspect |
| 67446 | Malignant neoplasm of lower lip, vermilion border |
| 101707 | Malignant neoplasm of lower lip, vermilion border NOS |
| 18678 | Malignant neoplasm of lower lobe bronchus |
| 12582 | Malignant neoplasm of lower lobe of lung |
| 31188 | Malignant neoplasm of lower lobe, bronchus or lung |
| 42566 | Malignant neoplasm of lower lobe, bronchus or lung NOS |
| 42416 | Malignant neoplasm of lower third of oesophagus |
| 59097 | Malignant neoplasm of lower uterine segment |
| 45222 | Malignant neoplasm of lower-inner quadrant of female breast |
| 42070 | Malignant neoplasm of lower-outer quadrant of female breast |
| 54691 | Malignant neoplasm of lumbar vertebra |
| 12323 | Malignant neoplasm of lymphatic and haemopoietic tissue |
| 12870 | Malignant neoplasm of main bronchus |
| 21698 | Malignant neoplasm of main bronchus NOS |
| 50475 | Malignant neoplasm of major salivary gland NOS |
| 20292 | Malignant neoplasm of major salivary glands |
| 59520 | Malignant neoplasm of malar bone |
| 19423 | Malignant neoplasm of male breast |
| 48809 | Malignant neoplasm of male breast NOS |
| 33833 | Malignant neoplasm of mandible |
| 71946 | Malignant neoplasm of mastoid air cells |
| 17475 | Malignant neoplasm of maxilla |
| 32174 | Malignant neoplasm of maxillary sinus |
| 63995 | Malignant neoplasm of Meckel's diverticulum |
| 34878 | Malignant neoplasm of medial cuneiform |
| 61064 | Malignant neoplasm of mediastinum, part unspecified |
| 49132 | Malignant neoplasm of medulla oblongata |
| 49875 | Malignant neoplasm of meninges, unspecified |
| 90290 | Malignant neoplasm of mesentery |
| 59388 | Malignant neoplasm of mesocaecum |
| 30165 | Malignant neoplasm of mesorectum |
| 72464 | Malignant neoplasm of metacarpal bones |
| 93537 | Malignant neoplasm of midbrain |
| 41523 | Malignant neoplasm of middle lobe bronchus |
| 39923 | Malignant neoplasm of middle lobe of lung |
| 31268 | Malignant neoplasm of middle lobe, bronchus or lung |
| 54134 | Malignant neoplasm of middle lobe, bronchus or lung NOS |
| 54171 | Malignant neoplasm of middle third of oesophagus |
| 107258 | Malignant neoplasm of midline of tongue |
| 55015 | Malignant neoplasm of mouth NOS |
| 103946 | Malignant neoplasm of mucosa of trachea |
| 65605 | Malignant neoplasm of myocardium |
| 45793 | Malignant neoplasm of myometrium of corpus uteri |
| 95458 | Malignant neoplasm of nasal bone |
| 23389 | Malignant neoplasm of nasal cavities |
| 42856 | Malignant neoplasm of nasal cavities NOS |
| 98911 | Malignant neoplasm of nasal conchae |
| 65357 | Malignant neoplasm of nasolacrimal duct |
| 106915 | Malignant neoplasm of nasopharyngeal soft palate surface |
| 24675 | Malignant neoplasm of nasopharynx |
| 28665 | Malignant neoplasm of nasopharynx NOS |
| 16280 | Malignant neoplasm of neck NOS |
| 56490 | Malignant neoplasm of nervous system NOS |
| 26853 | Malignant neoplasm of nipple and areola of female breast |
| 54494 | Malignant neoplasm of nipple and areola of male breast |
| 23380 | Malignant neoplasm of nipple of female breast |
| 68480 | Malignant neoplasm of nipple of male breast |
| 59831 | Malignant neoplasm of nipple or areola of female breast NOS |
| 12490 | Malignant neoplasm of nose NOS |
| 55953 | Malignant neoplasm of occipital bone |
| 39088 | Malignant neoplasm of occipital lobe |
| 1062 | Malignant neoplasm of oesophagus |
| 30700 | Malignant neoplasm of oesophagus NOS |
| 64971 | Malignant neoplasm of olfactory bulb |
| 50898 | Malignant neoplasm of omentum |
| 70126 | Malignant neoplasm of optic nerve |
| 45667 | Malignant neoplasm of orbit |
| 63104 | Malignant neoplasm of orbit NOS |
| 50298 | Malignant neoplasm of orbital bone |
| 22893 | Malignant neoplasm of oropharynx |
| 43200 | Malignant neoplasm of oropharynx NOS |
| 67323 | Malignant neoplasm of oropharynx, other specified sites |
| 45267 | Malignant neoplasm of other and ill defined site NOS |
| 9030 | Malignant neoplasm of other and ill-defined sites |
| 14792 | Malignant neoplasm of other and unspecified parts of mouth |
| 1056 | Malignant neoplasm of other and unspecified site NOS |
| 38736 | Malignant neoplasm of other and unspecified site OS |
| 10995 | Malignant neoplasm of other and unspecified sites |
| 70696 | Malignant neoplasm of other major salivary glands |
| 67949 | Malignant neoplasm of other male genital organ |
| 92329 | Malignant neoplasm of other male genital organ NOS |
| 100733 | Malignant neoplasm of other part of brain NOS |
| 71139 | Malignant neoplasm of other parts of brain |
| 32955 | Malignant neoplasm of other site of cervix |
| 43435 | Malignant neoplasm of other site of cervix NOS |
| 56715 | Malignant neoplasm of other site of female breast |
| 38475 | Malignant neoplasm of other site of female breast NOS |
| 54202 | Malignant neoplasm of other site of male breast |
| 29283 | Malignant neoplasm of other site of respiratory tract |
| 36949 | Malignant neoplasm of other site of urinary bladder |
| 97996 | Malignant neoplasm of other site of uterine adnexa |
| 31608 | Malignant neoplasm of other site of uterine body |
| 49758 | Malignant neoplasm of other sites lip, oral cavity, pharynx |
| 38961 | Malignant neoplasm of other sites of bronchus or lung |
| 56709 | Malignant neoplasm of other sites of floor of mouth |
| 101753 | Malignant neoplasm of other sites of gum |
| 41530 | Malignant neoplasm of other sites of tongue |
| 90659 | Malignant neoplasm of other specified endocrine gland |
| 95421 | Malignant neoplasm of other specified female genital organ |
| 88362 | Malignant neoplasm of other specified hypopharyngeal site |
| 37916 | Malignant neoplasm of other specified mouth parts |
| 88144 | Malignant neoplasm of other specified part of nervous system |
| 53591 | Malignant neoplasm of other specified part of oesophagus |
| 98104 | Malignant neoplasm of other specified pleura |
| 40437 | Malignant neoplasm of other specified site of eye |
| 55630 | Malignant neoplasm of other specified site of nasopharynx |
| 91037 | Malignant neoplasm of other specified site of oropharynx NOS |
| 55019 | Malignant neoplasm of other specified site of stomach |
| 65372 | Malignant neoplasm of other specified site of stomach NOS |
| 99896 | Malignant neoplasm of other specified site small intestine |
| 42218 | Malignant neoplasm of other specified sites |
| 48231 | Malignant neoplasm of other specified sites of colon |
| 48537 | Malignant neoplasm of other specified sites of pancreas |
| 18354 | Malignant neoplasm of other specified skin sites |
| 44884 | Malignant neoplasm of other urinary organs |
| 7805 | Malignant neoplasm of ovary |
| 19141 | Malignant neoplasm of ovary and other uterine adnexa |
| 36371 | Malignant neoplasm of overlapping lesion of bronchus & lung |
| 16967 | Malignant neoplasm of overlapping lesion of corpus uteri |
| 18882 | Malignant neoplasm of overlapping lesion of lip |
| 102151 | Malignant neoplasm of overlapping lesion of tonsil |
| 59286 | Malignant neoplasm of overlapping lesion of urinary organs |
| 27617 | Malignant neoplasm of overlapping lesion of vulva |
| 28559 | Malignant neoplasm of palate NOS |
| 70819 | Malignant neoplasm of palate unspecified |
| 101988 | Malignant neoplasm of palatine tonsil |
| 61510 | Malignant neoplasm of palatoglossal arch |
| 93842 | Malignant neoplasm of palatopharyngeal arch |
| 8166 | Malignant neoplasm of pancreas |
| 34388 | Malignant neoplasm of pancreas NOS |
| 35535 | Malignant neoplasm of pancreatic duct |
| 46153 | Malignant neoplasm of parametrium |
| 4218 | Malignant neoplasm of parathyroid gland |
| 72174 | Malignant neoplasm of paraurethral glands |
| 54747 | Malignant neoplasm of parietal bone |
| 19226 | Malignant neoplasm of parietal lobe |
| 64516 | Malignant neoplasm of parietal peritoneum |
| 67107 | Malignant neoplasm of parietal pleura |
| 4388 | Malignant neoplasm of parotid gland |
| 111779 | Malignant neoplasm of patella |
| 54631 | Malignant neoplasm of pelvic bones, sacrum and coccyx |
| 39413 | Malignant neoplasm of pelvic peritoneum |
| 52316 | Malignant neoplasm of pelvis |
| 55101 | Malignant neoplasm of pelvis NOS |
| 38938 | Malignant neoplasm of pelvis, sacrum or coccyx NOS |
| 63224 | Malignant neoplasm of penis and other male genital organ NOS |
| 3541 | Malignant neoplasm of penis and other male genital organs |
| 43392 | Malignant neoplasm of penis, part unspecified |
| 23480 | Malignant neoplasm of perianal skin |
| 94975 | Malignant neoplasm of pericardium |
| 65159 | Malignant neoplasm of perinephric tissue |
| 86046 | Malignant neoplasm of peripheral nerve of abdomen |
| 89258 | Malignant neoplasm of peripheral nerve of low limb, incl hip |
| 73988 | Malignant neoplasm of peripheral nerve of pelvis |
| 63695 | Malignant neoplasm of peripheral nerve of thorax |
| 61716 | Malignant neoplasm of peripheral nerve,upp limb,incl should |
| 63568 | Malignant neoplasm of peripheral nerves of head, face & neck |
| 58949 | Malignant neoplasm of phalanges of foot |
| 86812 | Malignant neoplasm of phalanges of hand |
| 37940 | Malignant neoplasm of pharyngeal recess |
| 46548 | Malignant neoplasm of pharyngeal tonsil |
| 16297 | Malignant neoplasm of pharynx unspecified |
| 42460 | Malignant neoplasm of pineal gland |
| 33271 | Malignant neoplasm of pinna NEC |
| 8550 | Malignant neoplasm of pituitary gland |
| 93762 | Malignant neoplasm of placenta |
| 31573 | Malignant neoplasm of pleura |
| 34742 | Malignant neoplasm of pleura NOS |
| 91240 | Malignant neoplasm of pons |
| 43548 | Malignant neoplasm of postcricoid region |
| 92720 | Malignant neoplasm of posterior mediastinum |
| 64462 | Malignant neoplasm of posterior pharynx |
| 69671 | Malignant neoplasm of posterior third of tongue |
| 95429 | Malignant neoplasm of posterior wall of nasopharynx |
| 96869 | Malignant neoplasm of posterior wall of nasopharynx NOS |
| 90124 | Malignant neoplasm of posterior wall of oropharynx |
| 96802 | Malignant neoplasm of posterior wall of stomach NEC |
| 42012 | Malignant neoplasm of posterior wall of urinary bladder |
| 50681 | Malignant neoplasm of prepuce (foreskin) |
| 48237 | Malignant neoplasm of prepylorus of stomach |
| 89916 | Malignant neoplasm of presacral region |
| 780 | Malignant neoplasm of prostate |
| 51921 | Malignant neoplasm of pubis |
| 19318 | Malignant neoplasm of pyloric antrum of stomach |
| 41215 | Malignant neoplasm of pyloric canal of stomach |
| 21620 | Malignant neoplasm of pylorus of stomach |
| 59092 | Malignant neoplasm of pylorus of stomach NOS |
| 39897 | Malignant neoplasm of pyriform sinus |
| 92371 | Malignant neoplasm of radius |
| 27855 | Malignant neoplasm of rectosigmoid junction |
| 1800 | Malignant neoplasm of rectum |
| 35357 | Malignant neoplasm of rectum, rectosigmoid junction and anus |
| 27540 | Malignant neoplasm of renal calyces |
| 12389 | Malignant neoplasm of renal pelvis |
| 54184 | Malignant neoplasm of renal pelvis NOS |
| 42569 | Malignant neoplasm of respiratory tract NOS |
| 28069 | Malignant neoplasm of retina |
| 24048 | Malignant neoplasm of retrocaecal tissue |
| 37724 | Malignant neoplasm of retromolar area |
| 21330 | Malignant neoplasm of retroperitoneum |
| 44108 | Malignant neoplasm of retroperitoneum and peritoneum |
| 16298 | Malignant neoplasm of retroperitoneum and peritoneum NOS |
| 61555 | Malignant neoplasm of retroperitoneum NOS |
| 37842 | Malignant neoplasm of rib |
| 51237 | Malignant neoplasm of rib, sternum and clavicle NOS |
| 27528 | Malignant neoplasm of ribs, sternum and clavicle |
| 69951 | Malignant neoplasm of roof of mouth |
| 94390 | Malignant neoplasm of roof of nasopharynx |
| 40966 | Malignant neoplasm of sacral vertebra |
| 107126 | Malignant neoplasm of sacrococcygeal region |
| 37165 | Malignant neoplasm of scalp |
| 54234 | Malignant neoplasm of scalp and skin of neck |
| 73760 | Malignant neoplasm of scalp or skin of neck NOS |
| 49054 | Malignant neoplasm of scapula |
| 71810 | Malignant neoplasm of scapula and long bones of upper arm |
| 47767 | Malignant neoplasm of scrotum |
| 37016 | Malignant neoplasm of sebaceous gland |
| 111426 | Malignant neoplasm of second metatarsal bone |
| 68161 | Malignant neoplasm of seminal vesicle |
| 62761 | Malignant neoplasm of septum of nose |
| 105475 | Malignant neoplasm of short bones of leg |
| 103354 | Malignant neoplasm of short bones of leg NOS |
| 2815 | Malignant neoplasm of sigmoid colon |
| 2492 | Malignant neoplasm of skin NOS |
| 18618 | Malignant neoplasm of skin of abdominal wall |
| 64270 | Malignant neoplasm of skin of ankle |
| 33997 | Malignant neoplasm of skin of auricle (ear) |
| 70380 | Malignant neoplasm of skin of axillary fold |
| 45077 | Malignant neoplasm of skin of back |
| 30543 | Malignant neoplasm of skin of breast |
| 62305 | Malignant neoplasm of skin of buttock |
| 30645 | Malignant neoplasm of skin of cheek, external |
| 37969 | Malignant neoplasm of skin of chest, excluding breast |
| 49403 | Malignant neoplasm of skin of chin |
| 62080 | Malignant neoplasm of skin of external auditory meatus |
| 55670 | Malignant neoplasm of skin of eyebrow |
| 25245 | Malignant neoplasm of skin of finger |
| 70587 | Malignant neoplasm of skin of foot |
| 30577 | Malignant neoplasm of skin of fore-arm |
| 30576 | Malignant neoplasm of skin of forehead |
| 67914 | Malignant neoplasm of skin of great toe |
| 66319 | Malignant neoplasm of skin of groin |
| 54352 | Malignant neoplasm of skin of hand |
| 104025 | Malignant neoplasm of skin of heel |
| 70988 | Malignant neoplasm of skin of hip |
| 56954 | Malignant neoplasm of skin of knee |
| 18245 | Malignant neoplasm of skin of lip |
| 33682 | Malignant neoplasm of skin of lower leg |
| 57442 | Malignant neoplasm of skin of lower limb and hip |
| 61194 | Malignant neoplasm of skin of lower limb or hip NOS |
| 43619 | Malignant neoplasm of skin of neck |
| 16202 | Malignant neoplasm of skin of nose (external) |
| 46458 | Malignant neoplasm of skin of perineum |
| 68197 | Malignant neoplasm of skin of popliteal fossa area |
| 66447 | Malignant neoplasm of skin of scapular region |
| 43122 | Malignant neoplasm of skin of shoulder |
| 21327 | Malignant neoplasm of skin of temple |
| 58601 | Malignant neoplasm of skin of thigh |
| 64406 | Malignant neoplasm of skin of thumb |
| 65782 | Malignant neoplasm of skin of toe |
| 57446 | Malignant neoplasm of skin of trunk, excluding scrotum |
| 15868 | Malignant neoplasm of skin of trunk, excluding scrotum, NOS |
| 67748 | Malignant neoplasm of skin of umbilicus |
| 42707 | Malignant neoplasm of skin of upper arm |
| 30747 | Malignant neoplasm of skin of upper limb and shoulder |
| 60526 | Malignant neoplasm of skin of upper limb or shoulder NOS |
| 6806 | Malignant neoplasm of small intestine and duodenum |
| 43390 | Malignant neoplasm of small intestine NOS |
| 40292 | Malignant neoplasm of soft palate |
| 40014 | Malignant neoplasm of soft tissue of face |
| 59382 | Malignant neoplasm of soft tissue of head |
| 48517 | Malignant neoplasm of soft tissue of neck |
| 46613 | Malignant neoplasm of specified parts of peritoneum |
| 64106 | Malignant neoplasm of specified parts of peritoneum NOS |
| 60052 | Malignant neoplasm of specified site NOS |
| 95783 | Malignant neoplasm of specified site of pancreas NOS |
| 63331 | Malignant neoplasm of spermatic cord |
| 55595 | Malignant neoplasm of sphenoid bone |
| 65215 | Malignant neoplasm of sphenoidal sinus |
| 105613 | Malignant neoplasm of sphincter of Oddi |
| 51115 | Malignant neoplasm of spinal cord |
| 49714 | Malignant neoplasm of spinal meninges |
| 67211 | Malignant neoplasm of spinal meninges NOS |
| 65460 | Malignant neoplasm of spleen NEC |
| 93778 | Malignant neoplasm of spleen NOS |
| 18619 | Malignant neoplasm of splenic flexure of colon |
| 57719 | Malignant neoplasm of squamocolumnar junction of cervix |
| 49491 | Malignant neoplasm of sternum |
| 8386 | Malignant neoplasm of stomach |
| 14800 | Malignant neoplasm of stomach NOS |
| 22441 | Malignant neoplasm of subglottis |
| 70928 | Malignant neoplasm of sublingual gland |
| 51786 | Malignant neoplasm of submandibular gland |
| 73510 | Malignant neoplasm of supraclavicular fossa NOS |
| 26165 | Malignant neoplasm of supraglottis |
| 40443 | Malignant neoplasm of sweat gland |
| 39870 | Malignant neoplasm of tail of pancreas |
| 95182 | Malignant neoplasm of talus |
| 49463 | Malignant neoplasm of tarsus of eyelid |
| 62104 | Malignant neoplasm of temporal bone |
| 46792 | Malignant neoplasm of temporal lobe |
| 47556 | Malignant neoplasm of temporal lobe NOS |
| 15148 | Malignant neoplasm of testis |
| 38510 | Malignant neoplasm of testis NOS |
| 62126 | Malignant neoplasm of thalamus |
| 69821 | Malignant neoplasm of the pouch of Douglas |
| 108638 | Malignant neoplasm of third metacarpal bone |
| 41362 | Malignant neoplasm of thoracic oesophagus |
| 32372 | Malignant neoplasm of thoracic vertebra |
| 47286 | Malignant neoplasm of thorax |
| 64810 | Malignant neoplasm of thorax NOS |
| 27483 | Malignant neoplasm of thymus |
| 62556 | Malignant neoplasm of thymus, heart and mediastinum |
| 47862 | Malignant neoplasm of thyroid cartilage |
| 5637 | Malignant neoplasm of thyroid gland |
| 40814 | Malignant neoplasm of tibia |
| 10283 | Malignant neoplasm of tongue |
| 40557 | Malignant neoplasm of tongue NOS |
| 37096 | Malignant neoplasm of tongue, junctional zone |
| 36161 | Malignant neoplasm of tongue, tip and lateral border |
| 16241 | Malignant neoplasm of tonsil |
| 24397 | Malignant neoplasm of tonsillar fossa |
| 100002 | Malignant neoplasm of tonsillar fossa NOS |
| 55066 | Malignant neoplasm of tonsillar pillar |
| 15221 | Malignant neoplasm of trachea |
| 37810 | Malignant neoplasm of trachea NOS |
| 13243 | Malignant neoplasm of trachea, bronchus and lung |
| 6935 | Malignant neoplasm of transverse colon |
| 38862 | Malignant neoplasm of trigone of urinary bladder |
| 67217 | Malignant neoplasm of trunk NOS |
| 47668 | Malignant neoplasm of tunica vaginalis |
| 96445 | Malignant neoplasm of turbinate |
| 54613 | Malignant neoplasm of tympanic antrum |
| 98537 | Malignant neoplasm of tympanic cavity |
| 64848 | Malignant neoplasm of ulna |
| 64602 | Malignant neoplasm of undescended testis |
| 96429 | Malignant neoplasm of undescended testis NOS |
| 47810 | Malignant neoplasm of unspecified site |
| 54267 | Malignant neoplasm of unspecified site NOS |
| 95772 | Malignant neoplasm of upper buccal sulcus |
| 55550 | Malignant neoplasm of upper eyelid |
| 32024 | Malignant neoplasm of upper gum |
| 27449 | Malignant neoplasm of upper limb NOS |
| 111289 | Malignant neoplasm of upper lip, buccal aspect |
| 66270 | Malignant neoplasm of upper lip, external |
| 99001 | Malignant neoplasm of upper lip, frenulum |
| 99493 | Malignant neoplasm of upper lip, inner aspect |
| 100721 | Malignant neoplasm of upper lip, inner aspect NOS |
| 50296 | Malignant neoplasm of upper lip, lipstick area |
| 98500 | Malignant neoplasm of upper lip, mucosa |
| 90610 | Malignant neoplasm of upper lip, oral aspect |
| 73962 | Malignant neoplasm of upper lip, vermilion border |
| 98740 | Malignant neoplasm of upper lip, vermilion border NOS |
| 31700 | Malignant neoplasm of upper lobe bronchus |
| 25886 | Malignant neoplasm of upper lobe of lung |
| 10358 | Malignant neoplasm of upper lobe, bronchus or lung |
| 44169 | Malignant neoplasm of upper lobe, bronchus or lung NOS |
| 50789 | Malignant neoplasm of upper third of oesophagus |
| 29826 | Malignant neoplasm of upper-inner quadrant of female breast |
| 23399 | Malignant neoplasm of upper-outer quadrant of female breast |
| 42023 | Malignant neoplasm of urachus |
| 15223 | Malignant neoplasm of ureter |
| 28241 | Malignant neoplasm of ureteric orifice |
| 101608 | Malignant neoplasm of ureteropelvic junction |
| 15644 | Malignant neoplasm of urethra |
| 779 | Malignant neoplasm of urinary bladder |
| 31102 | Malignant neoplasm of urinary bladder NOS |
| 65106 | Malignant neoplasm of uterine adnexa NOS |
| 2744 | Malignant neoplasm of uterus, part unspecified |
| 37516 | Malignant neoplasm of uvula |
| 37328 | Malignant neoplasm of vagina |
| 60772 | Malignant neoplasm of vagina NOS |
| 10698 | Malignant neoplasm of vaginal vault |
| 39554 | Malignant neoplasm of vallecula |
| 62840 | Malignant neoplasm of ventral surface of tongue |
| 38488 | Malignant neoplasm of ventral tongue surface NOS |
| 16704 | Malignant neoplasm of vertebral column |
| 49701 | Malignant neoplasm of vertebral column NOS |
| 103796 | Malignant neoplasm of vestibule of mouth |
| 62182 | Malignant neoplasm of vestibule of nose |
| 106194 | Malignant neoplasm of visceral pleura |
| 44452 | Malignant neoplasm of vomer |
| 4554 | Malignant neoplasm of vulva unspecified |
| 95016 | Malignant neoplasm of Waldeyer's ring |
| 54493 | Malignant neoplasm of xiphoid process |
| 50299 | Malignant neoplasm of zygomatic bone |
| 60312 | Malignant neoplasm other gallbladder/extrahepatic bile duct |
| 56918 | Malignant neoplasm other spec digestive tract and peritoneum |
| 42429 | Malignant neoplasm overlapping lesion of skin |
| 59823 | Malignant neoplasm pituitary gland and craniopharyngeal duct |
| 99386 | Malignant neoplasm posterior margin nasal septum and choanae |
| 50974 | Malignant neoplasm rectum,rectosigmoid junction and anus NOS |
| 53515 | Malignant neoplasm skin of ear and external auricular canal |
| 27370 | Malignant neoplasm skin of other and unspecified parts face |
| 46008 | Malignant neoplasm skin other and unspec part of face NOS |
| 108389 | Malignant neoplasm soft tissues of cervical spine |
| 53884 | Malignant neoplasm tonsil NOS |
| 111311 | Malignant neoplasm, overlap lesion connective & soft tissue |
| 66646 | Malignant neoplasm, overlap lesion of resp & intrathor orgs |
| 68824 | Malignant neoplasm, overlapping lesion male genital orgs |
| 39590 | Malignant neoplasm, overlapping lesion of accessory sinuses |
| 35039 | Malignant neoplasm, overlapping lesion of biliary tract |
| 47801 | Malignant neoplasm, overlapping lesion of bladder |
| 65241 | Malignant neoplasm, overlapping lesion of brain |
| 49148 | Malignant neoplasm, overlapping lesion of breast |
| 58094 | Malignant neoplasm, overlapping lesion of cervix uteri |
| 93478 | Malignant neoplasm, overlapping lesion of colon |
| 94776 | Malignant neoplasm, overlapping lesion of digestive system |
| 45922 | Malignant neoplasm, overlapping lesion of eye and adnexa |
| 17912 | Malignant neoplasm, overlapping lesion of floor of mouth |
| 50579 | Malignant neoplasm, overlapping lesion of larynx |
| 66422 | Malignant neoplasm, overlapping lesion of nasopharynx |
| 67497 | Malignant neoplasm, overlapping lesion of oesophagus |
| 97875 | Malignant neoplasm, overlapping lesion of pancreas |
| 52570 | Malignant neoplasm, overlapping lesion of penis |
| 66166 | Malignant neoplasm, overlapping lesion of small intestine |
| 51690 | Malignant neoplasm, overlapping lesion of stomach |
| 50777 | Malignant neoplasm,overlap lesion periph nerve & auton ns |
| 43614 | Malignant neoplasm/bones+articular cartilage/limb,unspfd |
| 67451 | Malignant neoplasm/overlap lesion/bone+articulr cartilage |
| 26454 | Malignant neoplasm/overlapping lesion/feml genital organs |
| 87113 | Malignant neoplasm-pluriglandular involvement,unspecified |
| 51352 | Malignant neoplasms of independent (primary) multiple sites |
| 65434 | Malignant neoplasms of lymphoid and histiocytic tissue NOS |
| 104324 | Malignant tumour of unknown origin |
| 65721 | Mast cell leukaemia |
| 57671 | Megakaryocytic leukaemia |
| 67700 | Monoblastic leukaemia |
| 35875 | Monocytic leukaemia |
| 93342 | Monocytic leukaemia NOS |
| 7176 | Myeloid leukaemia |
| 33344 | Myeloid leukaemia NOS |
| 20440 | Myelomonocytic leukaemia |
| 5179 | Nodular lymphoma (Brill - Symmers disease) |
| 65701 | Nodular lymphoma NOS |
| 92068 | Nodular lymphoma of intra-abdominal lymph nodes |
| 105203 | Nodular lymphoma of intrathoracic lymph nodes |
| 111766 | Nodular lymphoma of lymph nodes of axilla and upper limb |
| 45264 | Nodular lymphoma of lymph nodes of head, face and neck |
| 94995 | Nodular lymphoma of lymph nodes of inguinal region and leg |
| 58082 | Nodular lymphoma of lymph nodes of multiple sites |
| 66327 | Nodular lymphoma of unspecified site |
| 4865 | Oesophageal cancer |
| 95665 | Oestrogen receptor negative tumour |
| 95517 | Oestrogen receptor positive tumour |
| 94174 | Other and unspecified leukaemia |
| 99413 | Other and unspecified leukaemia NOS |
| 34692 | Other leukaemia of unspecified cell type |
| 49725 | Other lymphoid leukaemia |
| 38331 | Other lymphoid leukaemia NOS |
| 26034 | Other malignant neoplasm NOS |
| 33333 | Other malignant neoplasm of lymphoid and histiocytic tissue |
| 4632 | Other malignant neoplasm of skin |
| 99015 | Other monocytic leukaemia |
| 103645 | Other monocytic leukaemia NOS |
| 66089 | Other myeloid leukaemia NOS |
| 37272 | Other specified leukaemia |
| 30632 | Other specified leukaemia NOS |
| 90118 | Patient on regional cancer register |
| 39187 | Plasma cell leukaemia |
| 16126 | Primary carcinoma of liver |
| 25535 | Primary malignant neoplasm of liver |
| 44399 | Primary malignant neoplasm of liver NOS |
| 11035 | Primary malignant neoplasm of unknown site |
| 11991 | Primary vulval cancer |
| 103220 | Progesterone receptor negative tumour |
| 96284 | Progesterone receptor positive tumour |
| 31586 | Prolymphocytic leukaemia |
| 111627 | Prolymphocytic leukaemia of T-cell type |
| 46041 | Pseudotumour cerebri |
| 88762 | Radiological tumour control |
| 29285 | Radiotherapy-tumour palliation |
| 5901 | Rectal carcinoma |
| 94688 | Recurrence of tumour |
| 18712 | Renal malignant neoplasm |
| 94824 | Resection of heart tumour |
| 25366 | Secondary and unspec malig neop ant mediastinal lymph nodes |
| 44627 | Secondary and unspec malig neop anterior cervical LN |
| 50199 | Secondary and unspec malig neop axilla and upper limb LN |
| 73538 | Secondary and unspec malig neop axilla and upper limb LN NOS |
| 37540 | Secondary and unspec malig neop axillary lymph nodes |
| 62124 | Secondary and unspec malig neop bronchopulmonary lymph nodes |
| 101662 | Secondary and unspec malig neop circumflex iliac LN |
| 41691 | Secondary and unspec malig neop coeliac lymph nodes |
| 18658 | Secondary and unspec malig neop common iliac lymph nodes |
| 68611 | Secondary and unspec malig neop deep cervical LN |
| 61289 | Secondary and unspec malig neop deep inguinal lymph nodes |
| 92703 | Secondary and unspec malig neop deep parotid lymph nodes |
| 95378 | Secondary and unspec malig neop diaphragmatic lymph nodes |
| 69132 | Secondary and unspec malig neop external iliac lymph nodes |
| 61677 | Secondary and unspec malig neop inferior mesenteric LN |
| 69392 | Secondary and unspec malig neop inferior tracheobronchial LN |
| 50904 | Secondary and unspec malig neop infraclavicular lymph nodes |
| 63915 | Secondary and unspec malig neop inguinal and lower limb LN |
| 105953 | Secondary and unspec malig neop intercostal lymph nodes |
| 84368 | Secondary and unspec malig neop internal iliac lymph nodes |
| 37919 | Secondary and unspec malig neop internal mammary lymph nodes |
| 44931 | Secondary and unspec malig neop intra-abdominal LN NOS |
| 52736 | Secondary and unspec malig neop intra-abdominal lymph nodes |
| 72803 | Secondary and unspec malig neop intrapelvic LN NOS |
| 6701 | Secondary and unspec malig neop intrapelvic lymph nodes |
| 93716 | Secondary and unspec malig neop intrathoracic LN NOS |
| 64116 | Secondary and unspec malig neop intrathoracic lymph nodes |
| 49214 | Secondary and unspec malig neop lymph nodes head/face/neck |
| 20159 | Secondary and unspec malig neop lymph nodes multiple sites |
| 15507 | Secondary and unspec malig neop lymph nodes NOS |
| 28059 | Secondary and unspec malig neop of facial lymph nodes |
| 70747 | Secondary and unspec malig neop of inguinal and leg LN NOS |
| 64918 | Secondary and unspec malig neop of superficial parotid LN |
| 58692 | Secondary and unspec malig neop paratracheal lymph nodes |
| 46409 | Secondary and unspec malig neop pectoral lymph nodes |
| 55463 | Secondary and unspec malig neop post mediastinal lymph nodes |
| 52190 | Secondary and unspec malig neop pulmonary lymph nodes |
| 47366 | Secondary and unspec malig neop sacral lymph nodes |
| 39433 | Secondary and unspec malig neop submandibular lymph nodes |
| 38343 | Secondary and unspec malig neop submental lymph nodes |
| 67797 | Secondary and unspec malig neop superfic tracheobronchial LN |
| 33395 | Secondary and unspec malig neop superficial cervical LN |
| 54278 | Secondary and unspec malig neop superficial inguinal LN |
| 72713 | Secondary and unspec malig neop superficial mesenteric LN |
| 98626 | Secondary and unspec malig neop supratrochlear lymph nodes |
| 66775 | Secondary and unspec malignant neoplasm mastoid lymph nodes |
| 65253 | Secondary and unspec malignant neoplasm occipital lymph node |
| 9618 | Secondary and unspecified malignant neoplasm of lymph nodes |
| 97832 | Secondary cancer of the cervix |
| 65490 | Secondary cancer of the vulva |
| 27651 | Secondary carcinoma of other specified sites |
| 24301 | Secondary carcinoma of respiratory and/or digestive systems |
| 36200 | Secondary malig neop of large intestine or rectum NOS |
| 35053 | Secondary malig neop of respiratory and digestive systems |
| 66083 | Secondary malig neop of respiratory or digestive system NOS |
| 67396 | Secondary malig neop of retroperitoneum and peritoneum |
| 97672 | Secondary malig neop of retroperitoneum or peritoneum NOS |
| 70026 | Secondary malig neop of small intestine or duodenum NOS |
| 36401 | Secondary malignant neoplasm of adrenal gland |
| 22146 | Secondary malignant neoplasm of bladder |
| 7654 | Secondary malignant neoplasm of bone and bone marrow |
| 5198 | Secondary malignant neoplasm of brain |
| 33843 | Secondary malignant neoplasm of brain and spinal cord |
| 59375 | Secondary malignant neoplasm of brain or spinal cord NOS |
| 16760 | Secondary malignant neoplasm of breast |
| 73616 | Secondary malignant neoplasm of cervix uteri |
| 28727 | Secondary malignant neoplasm of colon |
| 55946 | Secondary malignant neoplasm of duodenum |
| 104480 | Secondary malignant neoplasm of epididymis and vas deferens |
| 99511 | Secondary malignant neoplasm of ileum |
| 110433 | Secondary malignant neoplasm of jejunum |
| 1952 | Secondary malignant neoplasm of kidney |
| 44529 | Secondary malignant neoplasm of large intestine and rectum |
| 36147 | Secondary malignant neoplasm of liver |
| 15103 | Secondary malignant neoplasm of liver |
| 4137 | Secondary malignant neoplasm of lung |
| 51551 | Secondary malignant neoplasm of mediastinum |
| 56345 | Secondary malignant neoplasm of other digestive organ |
| 54120 | Secondary malignant neoplasm of other part of nervous system |
| 62584 | Secondary malignant neoplasm of other respiratory organs |
| 16500 | Secondary malignant neoplasm of other specified site NOS |
| 22524 | Secondary malignant neoplasm of other specified site NOS |
| 5842 | Secondary malignant neoplasm of other specified sites |
| 18616 | Secondary malignant neoplasm of other specified sites |
| 62828 | Secondary malignant neoplasm of other urinary organ NOS |
| 73213 | Secondary malignant neoplasm of other urinary organs |
| 44615 | Secondary malignant neoplasm of ovary |
| 49145 | Secondary malignant neoplasm of penis |
| 27391 | Secondary malignant neoplasm of peritoneum |
| 16213 | Secondary malignant neoplasm of pleura |
| 21590 | Secondary malignant neoplasm of prostate |
| 62909 | Secondary malignant neoplasm of rectum |
| 35364 | Secondary malignant neoplasm of retroperitoneum |
| 19945 | Secondary malignant neoplasm of skin |
| 55096 | Secondary malignant neoplasm of skin NOS |
| 9505 | Secondary malignant neoplasm of skin of breast |
| 100296 | Secondary malignant neoplasm of skin of face |
| 43930 | Secondary malignant neoplasm of skin of head |
| 48828 | Secondary malignant neoplasm of skin of hip and leg |
| 35999 | Secondary malignant neoplasm of skin of neck |
| 63896 | Secondary malignant neoplasm of skin of shoulder and arm |
| 41144 | Secondary malignant neoplasm of skin of trunk |
| 64680 | Secondary malignant neoplasm of small intestine and duodenum |
| 38918 | Secondary malignant neoplasm of spinal cord |
| 34145 | Secondary malignant neoplasm of testis |
| 45824 | Secondary malignant neoplasm of tongue |
| 54679 | Secondary malignant neoplasm of unknown site |
| 60134 | Secondary malignant neoplasm of ureter |
| 53528 | Secondary malignant neoplasm of urethra |
| 55090 | Secondary malignant neoplasm of uterus |
| 70736 | Secondary malignant neoplasm of vagina |
| 60335 | Secondary malignant neoplasm of vulva |
| 67129 | Secondary unspec malig neop lymph nodes head/face/neck NOS |
| 98142 | Siewert type I adenocarcinoma |
| 97499 | Siewert type II adenocarcinoma |
| 96094 | Siewert type III adenocarcinoma |
| 54793 | Subacute leukaemia NOS |
| 72774 | Subacute lymphoid leukaemia |
| 101606 | Subacute monocytic leukaemia |
| 63475 | Subacute myeloid leukaemia |
| 104475 | Subacute myelomonocytic leukaemia |
| 107643 | T-cell prolymphocytic leukaemia |
| 65777 | Thrombocytic leukaemia |
| 67248 | Thyroid tumour/metast irradiat |
| 57122 | TNM tumour staging |
| 94676 | Tumour hormone receptor status |
| 104554 | Tumour lysis syndrome |
| 23224 | Tumour staging |
| 1949 | TURBT - transurethral resection of bladder tumour |
| 103353 | Unspec malig neop lymphoid/histiocytic intra-abdominal nodes |
| 107638 | Unspec malig neop lymphoid/histiocytic lymph node axilla/arm |
| 64427 | Unspec malig neop lymphoid/histiocytic lymph node head/neck |
| 71609 | Unspec malig neop lymphoid/histiocytic nodes inguinal/leg |
| 109342 | Unspec malig neop lymphoid/histiocytic of intrapelvic nodes |
| 93384 | Unspec malig neop lymphoid/histiocytic of intrathoracic node |
| 101465 | Unspec malig neop lymphoid/histiocytic of multiple sites |
| 108037 | Unspec malig neop lymphoid/histiocytic of unspecified site |
| 72215 | Uterine body tumour in pregnancy/childbirth/puerperium NOS |
| 108922 | Wilms' tumour + nephrotic syndrome + pseudohermaphroditism |

**Supplementary Table 1b. CPRD codelist for specific types of cancer**

| **Cancer types** | **Medcode** | **Description** |
| --- | --- | --- |
| Breast cancer | 12480 | [M]Paget's disease and intraductal carcinoma of breast |
|  | 40359 | [M]Juvenile breast carcinoma |
|  | 67701 | [M]Secretory breast carcinoma |
|  | 9902 | Carcinoma of bone, connective tissue, skin and breast |
|  | 42542 | [M]Paget's disease and infiltrating breast duct carcinoma |
|  | 42070 | Malignant neoplasm of lower-outer quadrant of female breast |
|  | 45222 | Malignant neoplasm of lower-inner quadrant of female breast |
|  | 31546 | Malignant neoplasm of central part of female breast |
|  | 16639 | [V]Personal history of malignant neoplasm of breast |
|  | 56715 | Malignant neoplasm of other site of female breast |
|  | 54494 | Malignant neoplasm of nipple and areola of male breast |
|  | 9505 | Secondary malignant neoplasm of skin of breast |
|  | 54202 | Malignant neoplasm of other site of male breast |
|  | 38475 | Malignant neoplasm of other site of female breast NOS |
|  | 48809 | Malignant neoplasm of male breast NOS |
|  | 95323 | Malignant neoplasm of ectopic site of male breast |
|  | 68480 | Malignant neoplasm of nipple of male breast |
|  | 59831 | Malignant neoplasm of nipple or areola of female breast NOS |
|  | 67884 | Malignant neoplasm of areola of male breast |
|  | 12499 | [X]Malignant neoplasm of breast |
|  | 64686 | Malignant neoplasm of areola of female breast |
|  | 30543 | Malignant neoplasm of skin of breast |
|  | 20685 | Malignant neoplasm of axillary tail of female breast |
|  | 16760 | Secondary malignant neoplasm of breast |
|  | 9470 | Malignant neoplasm of female breast NOS |
|  | 23399 | Malignant neoplasm of upper-outer quadrant of female breast |
|  | 29826 | Malignant neoplasm of upper-inner quadrant of female breast |
|  | 26853 | Malignant neoplasm of nipple and areola of female breast |
|  | 23380 | Malignant neoplasm of nipple of female breast |
|  | 19423 | Malignant neoplasm of male breast |
|  | 49148 | Malignant neoplasm, overlapping lesion of breast |
|  | 95057 | Malignant neoplasm of ectopic site of female breast |
|  | 3968 | Malignant neoplasm of female breast |
| Prostate cancer | 21590 | Secondary malignant neoplasm of prostate |
|  | 37306 | [V]Personal history of malignant neoplasm of prostate |
|  | 780 | Malignant neoplasm of prostate |
|  | 102314 | H/O: prostate cancer |
|  | 10178 | Gleason grading of prostate cancer |
|  | 21590 | Secondary malignant neoplasm of prostate |
|  | 37306 | [V]Personal history of malignant neoplasm of prostate |
|  | 780 | Malignant neoplasm of prostate |
|  | 102314 | H/O: prostate cancer |
|  | 10178 | Gleason grading of prostate cancer |
| Lung cancer | 35325 | [X]Malignant neoplasm of respiratory and intrathoracic orga |
|  | 22156 | ]Malignant tumour, small cell type |
|  | 45307 | Carcinoma of respiratory tract and intrathoracic organs |
|  | 35474 | Giant cell carcinoma |
|  | 2587 | Lung cancer |
|  | 34075 | Malig neop of respiratory tract and intrathoracic organs |
|  | 65793 | Malig neop of upper respiratory tract, part unspecified |
|  | 40595 | Malignant neoplasm of bronchus or lung |
|  | 3903 | Malignant neoplasm of bronchus or lung |
|  | 17391 | Malignant neoplasm of carina of bronchus |
|  | 33444 | Malignant neoplasm of hilus of lung |
|  | 18678 | Malignant neoplasm of lower lobe bronchus |
|  | 12582 | Malignant neoplasm of lower lobe of lung |
|  | 31188 | Malignant neoplasm of lower lobe, bronchus or lung |
|  | 42566 | Malignant neoplasm of lower lobe, bronchus or lung NOS |
|  | 12870 | Malignant neoplasm of main bronchus |
|  | 21698 | Malignant neoplasm of main bronchus NOS |
|  | 41523 | Malignant neoplasm of middle lobe bronchus |
|  | 39923 | Malignant neoplasm of middle lobe of lung |
|  | 31268 | Malignant neoplasm of middle lobe, bronchus or lung |
|  | 54134 | Malignant neoplasm of middle lobe, bronchus or lung NOS |
|  | 29283 | Malignant neoplasm of other site of respiratory tract |
|  | 36371 | Malignant neoplasm of overlapping lesion of bronchus & lung |
|  | 42569 | Malignant neoplasm of respiratory tract NOS |
|  | 15221 | Malignant neoplasm of trachea |
|  | 13243 | Malignant neoplasm of trachea, bronchus and lung |
|  | 31700 | Malignant neoplasm of upper lobe bronchus |
|  | 25886 | Malignant neoplasm of upper lobe of lung |
|  | 10358 | Malignant neoplasm of upper lobe, bronchus or lung |
|  | 44169 | Malignant neoplasm of upper lobe, bronchus or lung NOS |
|  | 21715 | Mesothelioma of lung |
|  | 9156 | Oat cell carcinoma |
|  | 20170 | Pancoast's syndrome |
|  | 35053 | Secondary malig neop of respiratory and digestive systems |
|  | 66083 | Secondary malig neop of respiratory or digestive system NOS |
|  | 4137 | Secondary malignant neoplasm of lung |
|  | 9291 | Small cell carcinoma NOS |
|  | 30988 | Small cell carcinoma, intermediate cell |
|  | 21217 | Small cell-large cell carcinoma |
|  | 29284 | [V]Personal history of malignant neoplasm of lung |
|  | 38961 | Malignant neoplasm of other sites of bronchus or lung |
|  | 49289 | [V]Personal history of malig neop of trachea/bronchus/lung |
|  | 32246 | [V]Personal history of malignant neoplasm of bronchus |
| Colorectal cancer | 4170 | [M]Adenomatous and adenocarcinomatous polyps of colon |
|  | 9491 | Anal carcinoma |
|  | 11628 | Cancer of bowel |
|  | 22163 | Carcinoma of caecum |
|  | 7219 | Carcinoma of rectum |
|  | 9118 | Colonic cancer |
|  | 101700 | Hereditary nonpolyposis colon cancer |
|  | 24370 | Malignant neoplasm of anal canal |
|  | 27897 | Malignant neoplasm of anus unspecified |
|  | 10946 | Malignant neoplasm of ascending colon |
|  | 3811 | Malignant neoplasm of caecum |
|  | 1220 | Malignant neoplasm of colon |
|  | 28163 | Malignant neoplasm of colon NOS |
|  | 10864 | Malignant neoplasm of descending colon |
|  | 9088 | Malignant neoplasm of hepatic flexure of colon |
|  | 27855 | Malignant neoplasm of rectosigmoid junction |
|  | 1800 | Malignant neoplasm of rectum |
|  | 35357 | Malignant neoplasm of rectum, rectosigmoid junction and anus |
|  | 2815 | Malignant neoplasm of sigmoid colon |
|  | 6935 | Malignant neoplasm of transverse colon |
|  | 5901 | Rectal carcinoma |
|  | 28727 | Secondary malignant neoplasm of colon |
|  | 44529 | Secondary malignant neoplasm of large intestine and rectum |
|  | 62909 | Secondary malignant neoplasm of rectum |
|  | 18619 | Malignant neoplasm of splenic flexure of colon |
|  | 39875 | [M]Adenomatous or adenocarcinomatous polyps of the colon NOS |
|  | 41702 | [M]Adenomatous and adenocarcinomatous polyps of colon |
|  | 93478 | Malignant neoplasm, overlapping lesion of colon |
|  | 48231 | Malignant neoplasm of other specified sites of colon |
|  | 94000 | Bowel cancer detected by national screening programme |
|  | 108905 | Bowel scope (flexible sigmoidoscopy) screen: cancer detected |
|  | 62785 | [V]Personal history of malignant neoplasm of rectum |
|  | 30165 | Malignant neoplasm of mesorectum |
|  | 50974 | Malignant neoplasm rectum,rectosigmoid junction and anus NOS |
|  | 44529 | Secondary malignant neoplasm of large intestine and rectum |
|  | 57727 | [V]Personal history of malignant neoplasm of large intestine |
|  | 6806 | Malignant neoplasm of small intestine and duodenum |
|  | 43390 | Malignant neoplasm of small intestine NOS |
|  | 99896 | Malignant neoplasm of other specified site small intestine |
|  | 66166 | Malignant neoplasm, overlapping lesion of small intestine |
|  | 64680 | Secondary malignant neoplasm of small intestine and duodenum |
| Melanoma | 56121 | [X]Malignant neoplasm of skin, unspecified |
|  | 24375 | Dermatofibrosarcoma protuberans |
|  | 57336 | Epithelioma, malignant |
|  | 19041 | Intraepidermal carcinoma NOS |
|  | 27931 | Kaposi's sarcoma of skin |
|  | 37618 | Malignant neoplasm of axilla NOS |
|  | 20685 | Malignant neoplasm of axillary tail of female breast |
|  | 23480 | Malignant neoplasm of perianal skin |
|  | 37165 | Malignant neoplasm of scalp |
|  | 54234 | Malignant neoplasm of scalp and skin of neck |
|  | 2492 | Malignant neoplasm of skin NOS |
|  | 33997 | Malignant neoplasm of skin of auricle of ear |
|  | 45077 | Malignant neoplasm of skin of back |
|  | 30543 | Malignant neoplasm of skin of breast |
|  | 30645 | Malignant neoplasm of skin of cheek, external |
|  | 37969 | Malignant neoplasm of skin of chest, excluding breast |
|  | 49403 | Malignant neoplasm of skin of chin |
|  | 25245 | Malignant neoplasm of skin of finger |
|  | 70587 | Malignant neoplasm of skin of foot |
|  | 30577 | Malignant neoplasm of skin of fore-arm |
|  | 30576 | Malignant neoplasm of skin of forehead |
|  | 54352 | Malignant neoplasm of skin of hand |
|  | 18245 | Malignant neoplasm of skin of lip |
|  | 33682 | Malignant neoplasm of skin of lower leg |
|  | 57442 | Malignant neoplasm of skin of lower limb and hip |
|  | 43619 | Malignant neoplasm of skin of neck |
|  | 16202 | Malignant neoplasm of skin of nose (external) |
|  | 43122 | Malignant neoplasm of skin of shoulder |
|  | 21327 | Malignant neoplasm of skin of temple |
|  | 57446 | Malignant neoplasm of skin of trunk, excluding scrotum |
|  | 15868 | Malignant neoplasm of skin of trunk, excluding scrotum, NOS |
|  | 67748 | Malignant neoplasm of skin of umbilicus |
|  | 42707 | Malignant neoplasm of skin of upper arm |
|  | 30747 | Malignant neoplasm of skin of upper limb and shoulder |
|  | 60526 | Malignant neoplasm of skin of upper limb or shoulder NOS |
|  | 53515 | Malignant neoplasm skin of ear and external suricular canal |
|  | 27370 | Malignant neoplasm skin of other and unspecified parts face |
|  | 4632 | Other malignant neoplasm of skin |
|  | 19945 | Secondary malignant neoplasm of skin |
|  | 55096 | Secondary malignant neoplasm of skin NOS |
|  | 9505 | Secondary malignant neoplasm of skin of Breast |
|  | 43930 | Secondary malignant neoplasm of skin of head |
|  | 48828 | Secondary malignant neoplasm of skin of hip and leg |
|  | 41144 | Secondary malignant neoplasm of skin of trunk |
|  | 60162 | [X]Malignant neoplasm overlapping lesion of skin |

**Supplementary Table 2. CPRD codelist for dementia diagnosis and drug prescription**

| **Sources** | **Code** | **Description** |
| --- | --- | --- |
| Dementia diagnosis (Medcode) | 4693 | [X] Unspecified dementia |
|  | 6578 | Vascular dementia |
|  | 7323 | Uncomplicated senile dementia |
|  | 7572 | Lewy body dementia |
|  | 8934 | [X]Subcortical vascular dementia |
|  | 11175 | Multi-infarct dementia |
|  | 18386 | Senile dementia with paranoia |
|  | 19393 | [X]Vascular dementia, unspecified |
|  | 19477 | Arteriosclerotic dementia |
|  | 21887 | Senile dementia with depression |
|  | 26270 | [X]Lewy body dementia |
|  | 29512 | Senile degeneration of brain |
|  | 31016 | [X]Mixed cortical and subcortical vascular dementia |
|  | 37015 | Senile dementia with delirium |
|  | 41089 | Senile dementia with depressive or paranoid features NOS |
|  | 42279 | Arteriosclerotic dementia NOS |
|  | 43089 | Uncomplicated arteriosclerotic dementia |
|  | 43292 | Arteriosclerotic dementia with depression |
|  | 44674 | Senile dementia with depressive or paranoid features |
|  | 46488 | [X]Vascular dementia of acute onset |
|  | 53446 | [X]Delirium superimposed on dementia |
|  | 55313 | Other vascular dementia |
|  | 55467 | Arteriosclerotic dementia with paranoia |
|  | 56912 | Arteriosclerotic dementia with delirium |
|  | 64267 | [X]Dementia in other specified diseases classif elsewhere |
|  | 5931 | H/O: dementia |
|  | 6061 | Organic memory impairment |
|  | 1916 | Senile dementia |
|  | 1350 | Senile/presenile dementia |
|  | 1917 | Alzheimer’s disease |
|  | 7664 | [X]Dementia in Alzheimer's disease |
|  | 16797 | Alzheimer's disease with early onset |
|  | 29386 | [X]Dementia in Alzheimer's disease, unspecified |
|  | 30706 | [X]Dementia in Alzheimer's dis, atypical or mixed type |
|  | 32057 | Alzheimer's disease with late onset |
|  | 38678 | [X]Dementia in Alzheimer's disease with late onset |
|  | 49263 | [X]Dementia in Alzheimer's disease with early onset |
|  | 59122 | [X]Other Alzheimer's disease |
|  | 60059 | [X]Primary degen dementia, Alzheimer's type, presenile onset |
|  | 61528 | [X]Alzheimer's disease type 2 |
|  | 46762 | [X]Alzheimer's disease type 1 |
| Dementia-specific drugs (product code) |  |  |
| Rivastigmine | 11751 | Rivastigmine 3mg capsules |
|  | 57627 | Rivatev 4.6mg/24hours transdermal patches (Teva UK Ltd) |
|  | 57171 | Erastig 9.5mg/24hours transdermal patches (Teva UK Ltd) |
|  | 55928 | Exelon 4.5mg capsules (Waymade Healthcare Plc) |
|  | 36976 | Rivastigmine 4.6mg/24hours transdermal patches |
|  | 56771 | Rivastigmine 3mg capsules (Dr Reddy's Laboratories (UK) Ltd) |
|  | 11546 | Exelon 1.5mg capsules (Novartis Pharmaceuticals UK Ltd) |
|  | 5616 | Exelon 6mg capsules (Novartis Pharmaceuticals UK Ltd) |
|  | 60723 | Rivastigmine 6mg capsules (Waymade Healthcare Plc) |
|  | 58780 | Voleze 9.5mg/24hours transdermal patches (Focus Pharmaceuticals Ltd) |
|  | 11752 | Rivastigmine 4.5mg capsules |
|  | 53882 | Rivastigmine 2mg/ml oral solution |
|  | 37957 | Exelon 9.5mg/24hours transdermal patches (Novartis Pharmaceuticals UK Ltd) |
|  | 11827 | Rivastigmine 2mg/ml oral solution sugar free |
|  | 37444 | Exelon 4.6mg/24hours transdermal patches (Novartis Pharmaceuticals UK Ltd) |
|  | 11716 | Exelon 3mg capsules (Novartis Pharmaceuticals UK Ltd) |
|  | 4597 | Rivastigmine 1.5mg capsules |
|  | 9786 | Rivastigmine 6mg capsules |
|  | 20404 | Exelon 4.5mg capsules (Novartis Pharmaceuticals UK Ltd) |
|  | 37132 | Rivastigmine 9.5mg/24hours transdermal patches |
|  | 18556 | Exelon 2mg/ml oral solution (Novartis Pharmaceuticals UK Ltd) |
|  | 58937 | Exelon 13.3mg/24hours transdermal patches (Novartis Pharmaceuticals UK Ltd) |
| Galantamine | 7329 | Galantamine 20mg/5ml oral solution sugar free |
|  | 5334 | Reminyl 12mg tablets (Shire Pharmaceuticals Ltd) |
|  | 10255 | Galantamine 8mg modified-release capsules |
|  | 61476 | Acumor XL 24mg capsules (Generics (UK) Ltd) |
|  | 24088 | Reminyl XL 24mg capsules (Shire Pharmaceuticals Ltd) |
|  | 11635 | Galantamine 12mg tablets |
|  | 9854 | Reminyl 4mg tablets (Shire Pharmaceuticals Ltd) |
|  | 18587 | Reminyl XL 8mg capsules (Shire Pharmaceuticals Ltd) |
|  | 11654 | Galantamine 8mg tablets |
|  | 48482 | Galsya XL 8mg capsules (Consilient Health Ltd) |
|  | 55720 | Gatalin XL 24mg capsules (Aspire Pharma Ltd) |
|  | 10187 | Galantamine 4mg tablets |
|  | 20140 | Reminyl XL 16mg capsules (Shire Pharmaceuticals Ltd) |
|  | 60493 | Galantex XL 24mg capsules (Creo Pharma Ltd) |
|  | 56709 | Gatalin XL 16mg capsules (Aspire Pharma Ltd) |
|  | 7361 | Galantamine 24mg modified-release capsules |
|  | 29288 | Reminyl 4mg/ml oral solution (Shire Pharmaceuticals Ltd) |
|  | 14309 | Galantamine 16mg modified-release capsules |
|  | 48015 | Galsya XL 24mg capsules (Consilient Health Ltd) |
|  | 18062 | Reminyl 8mg tablets (Shire Pharmaceuticals Ltd) |
| Donepezil | 56600 | Donepezil 5mg tablets (Zentiva) |
|  | 37188 | Aricept Evess 10mg orodispersible tablets (Eisai Ltd) |
|  | 2931 | Donepezil 10mg tablets |
|  | 35088 | Donepezil 10mg orodispersible tablets sugar free |
|  | 60107 | Donepezil 5mg tablets (Alliance Healthcare (Distribution) Ltd) |
|  | 53842 | Aricept 5mg tablets (Waymade Healthcare Plc) |
|  | 36848 | Aricept Evess 5mg orodispersible tablets (Eisai Ltd) |
|  | 59871 | Donepezil 10mg/5ml oral suspension |
|  | 2930 | Donepezil 5mg tablets |
|  | 35179 | Donepezil 5mg orodispersible tablets sugar free |
|  | 5247 | Aricept 10mg tablets (Eisai Ltd) |
|  | 5400 | Aricept 5mg tablets (Eisai Ltd) |
|  | 58947 | Donepezil 10mg tablets (Accord Healthcare Ltd) |
|  | 58709 | Donepezil 10mg tablets (A A H Pharmaceuticals Ltd) |
| Memantine | 9966 | Ebixa 5mg/pump oral solution (Lundbeck Ltd) |
|  | 38976 | Memantine 5mg+10mg+15mg+20mg Tablet |
|  | 61385 | Nemdatine 10mg tablets (Actavis UK Ltd) |
|  | 6225 | Memantine 10mg tablets |
|  | 39240 | Memantine 20mg tablets |
|  | 57139 | Ebixa 10mg tablets (DE Pharmaceuticals) |
|  | 61618 | Nemdatine 20mg tablets (Actavis UK Ltd) |
|  | 39363 | Ebixa 20mg tablets (Lundbeck Ltd) |
|  | 18800 | Ebixa 10mg tablets (Lundbeck Ltd) |
|  | 39362 | Ebixa tablets treatment initiation pack (Lundbeck Ltd) |
|  | 11837 | Memantine 10mg/ml oral solution sugar free |

**Supplementary Table 3. Sensitivity analyses of association between cancer and risk of dementia incidence**

| **Sensitivity analyses** | **N** | **Hazard ratios (95% CI)** | | |
| --- | --- | --- | --- | --- |
|  |  | **Model 1** | **Model 2** |  |
| Restricted to those who were at least 70 years old at cohort entry | 1,908,024 | 0.76 (0.75-0.77) | 0.77 (0.76-0.78) |  |
| Restricted to those who entered the cohort after year 2000 | 1,460,867 | 0.88 (0.85-0.90) | 0.86 (0.84-0.89) |  |
| Restricted to those who survived to at least 80 years old | 2,731,197 | 0.81 (0.80-0.82) | 0.78 (0.77-0.79) |  |
| Restricted to those who did not develop dementia within the first two years of follow-up | 3,002,661 | 0.77 (0.76-0.78) | 0.74 (0.73-0.75) |  |
| Complete case analysis | 1,337,876 | 0.80 (0.78-0.81) | 0.80 (0.78-0.82) |  |

Note: Non-cancer group was used as the reference group in Cox models. Model 1: adjust for gender, calendar year (time-varying), region. Model 2: model 1 + IMD, smoking status, BMI category. Model 3: model 2 + diabetes and cardiovascular diseases.

**Supplementary Table 4. Sensitivity analyses of Mendelian randomization analysis on overall and subtypes of cancer and risk of Alzheimer’s disease**

| Exposure | Number of SNPs | OR (95% CI) | | | |
| --- | --- | --- | --- | --- | --- |
|  |  | **MR-Egger regression** | **Maximum likelihood** | **Weighted median** | **Weighted mode** |
| Breast cancer | 172 | 0.93 (0.85-1.01) | 0.94 (0.90-0.98) | 0.92 (0.86-0.98) | 0.92 (0.84-1.02) |
| Prostate cancer | 103 | 0.95 (0.87-1.04) | 0.97 (0.94-1.01) | 1.00 (0.94-1.06) | 1.00 (0.92-1.09) |
| Lung cancer | 14 | 0.80 (0.68-0.93) | 0.90 (0.84-0.96) | 0.88 (0.79-0.97) | 0.87 (0.78-0.96) |
| Colorectal cancer | 73 | 1.01 (0.89-1.14) | 1.00 (0.96-1.04) | 1.02 (0.96-1.08) | 1.02 (0.93-1.11) |
| Melanoma | 63 | 0.97 (0.90-1.04) | 0.98 (0.95-1.02) | 0.93 (0.87-0.99) | 0.90 (0.82-1.00) |
| Overall cancer | 407 | 0.96 (0.92-0.99) | 0.97 (0.95-0.99) | 0.98 (0.95-1.01) | 0.93 (0.87-1.00) |

Note: OR = odds ratio; CI = confidence interval.

**Supplementary Figure 1. Forest plots of Mendelian randomization analysis on overall and subtypes of cancer and risk of Alzheimer’s disease**

1. **Overall cancer**


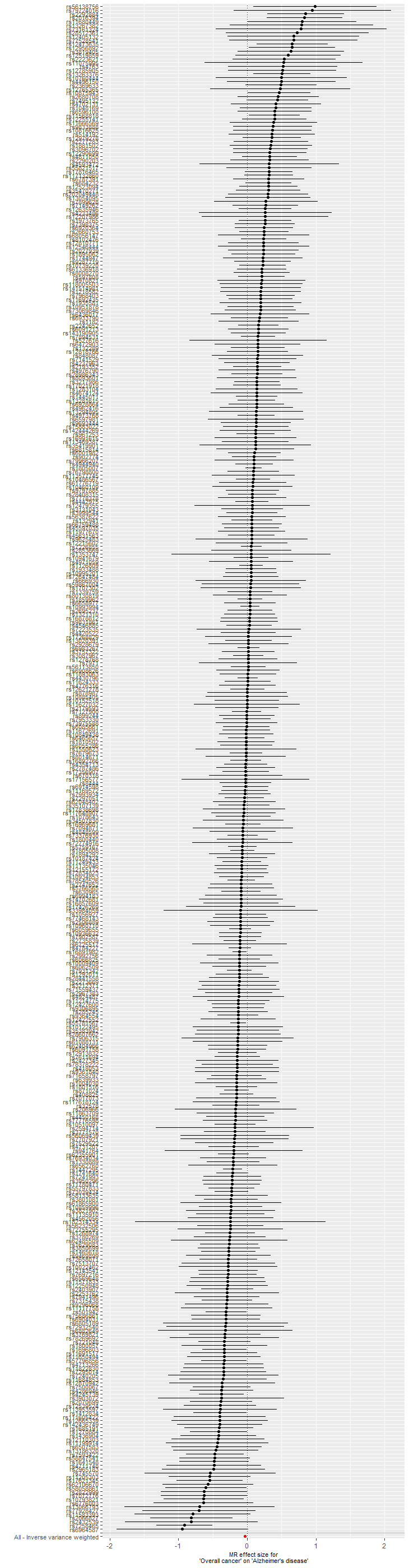


**b. Breast cancer**


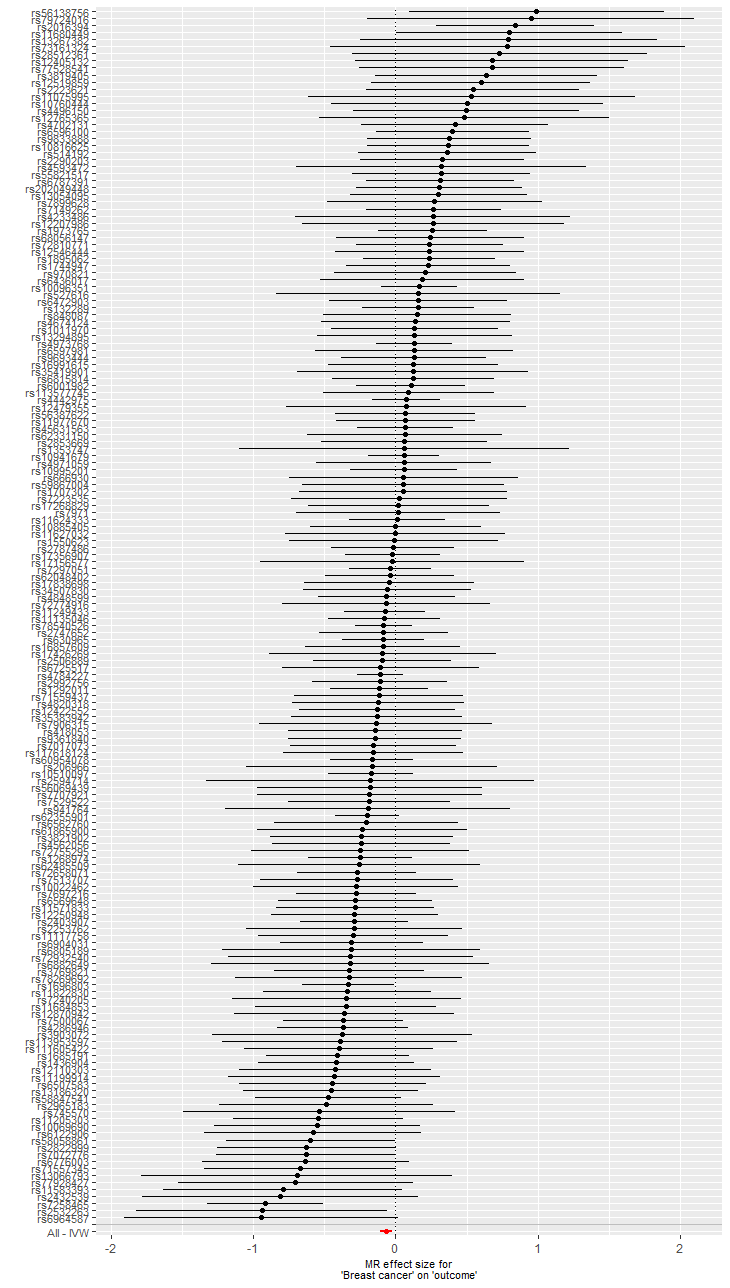


**c. Prostate cancer**


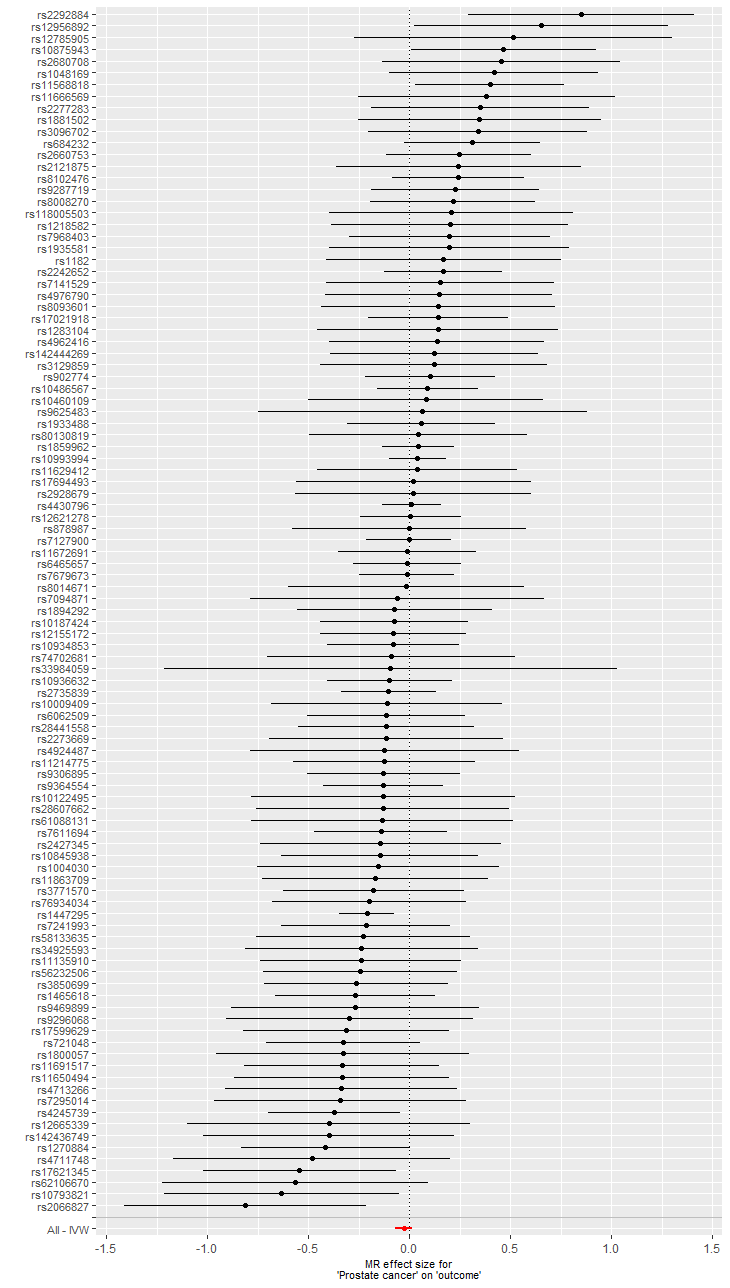


**d. Lung cancer**


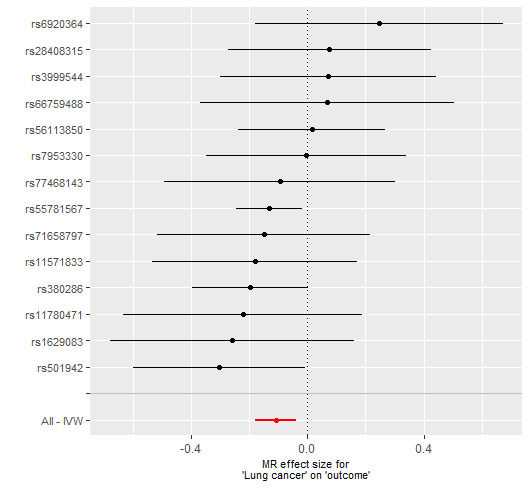


**e. Colorectal cancer**


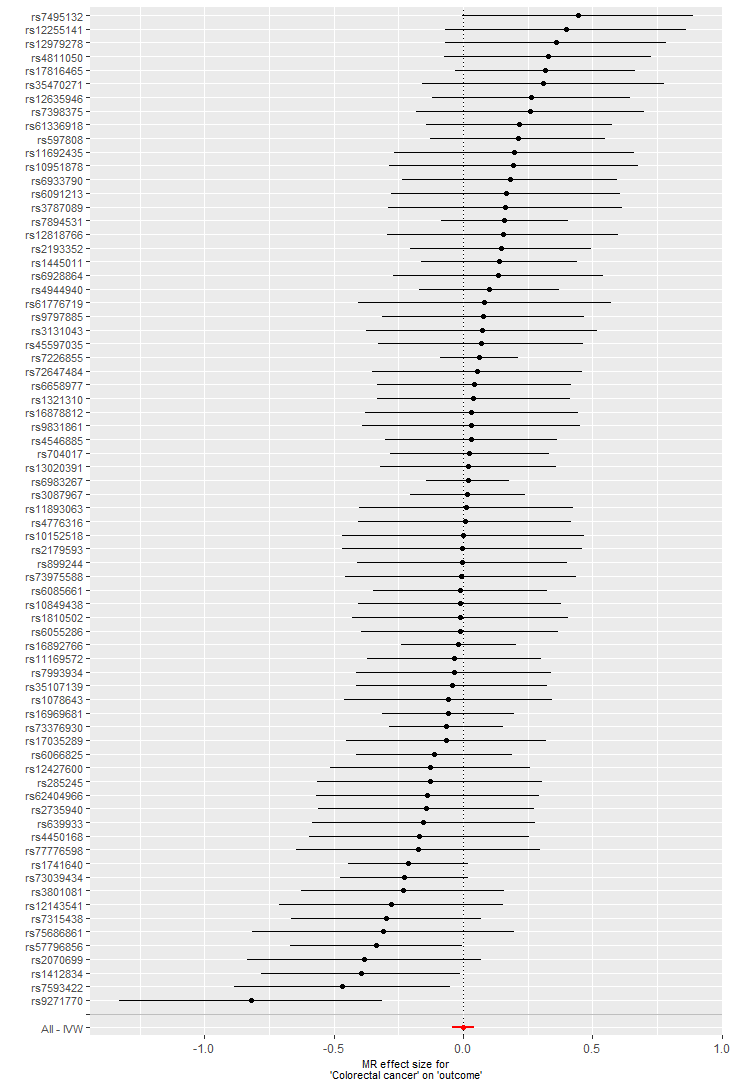


**f. Melanoma**


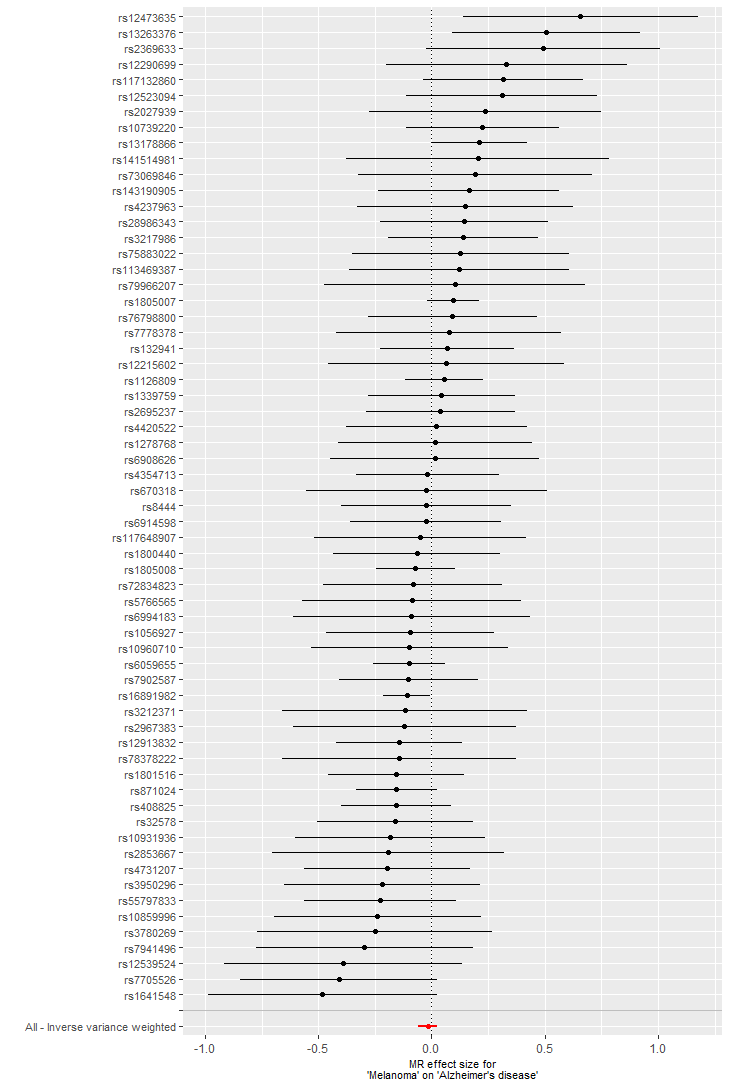


**Supplementary Figure 2. Funnel plots of Mendelian randomization analysis on overall and subtypes of cancer and risk of Alzheimer’s disease**

1. **Overall cancer**

**
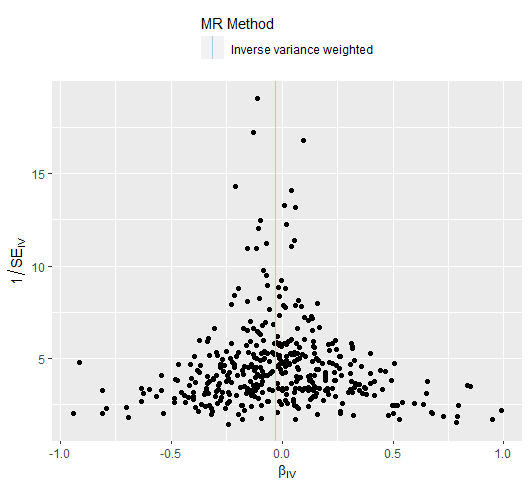
**

1. **Breast cancer**


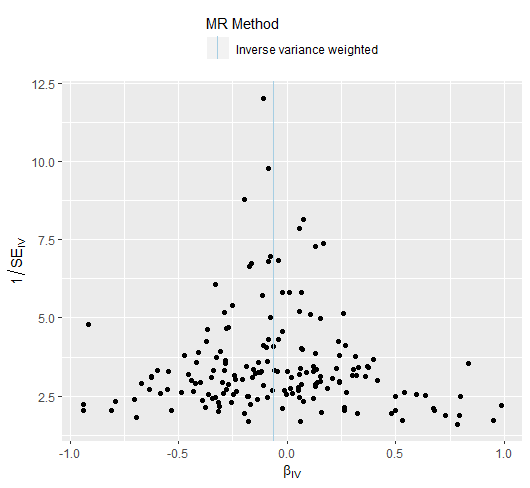


1. **Prostate cancer**


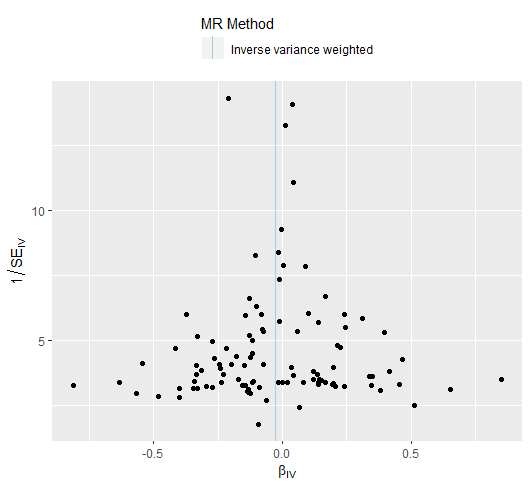


1. **Lung cancer**


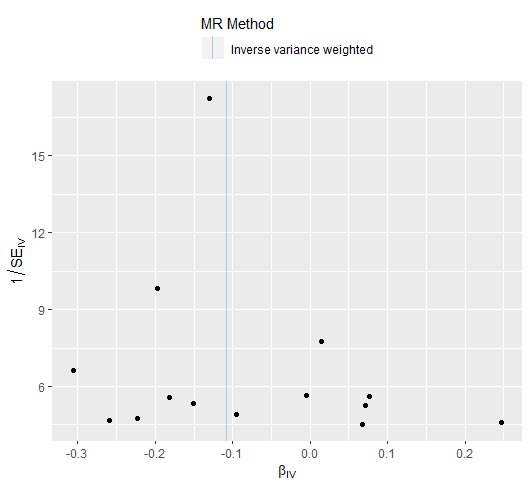


1. **Colorectal cancer**


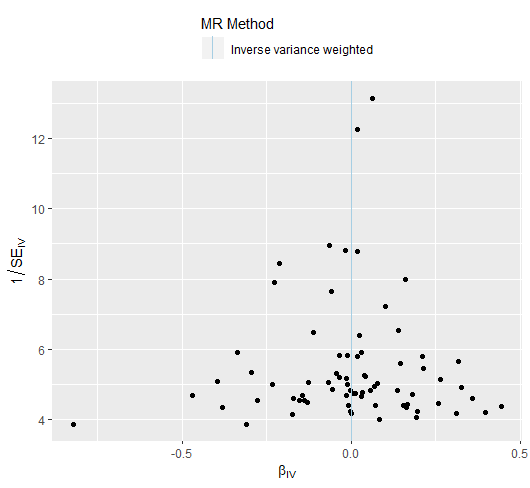


1. **Melanoma**


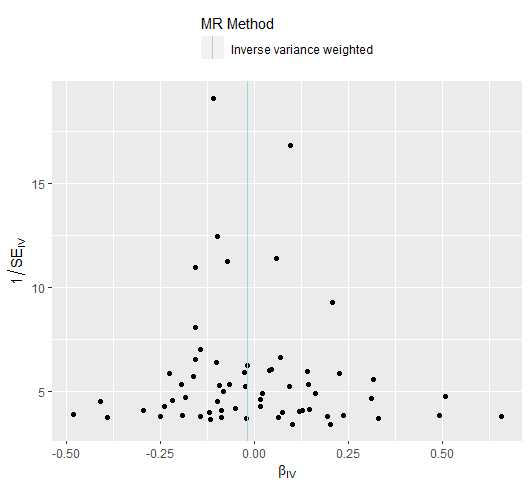

Supplement: Supplementary file 1 — Supplementary material, approximately 348 KB. [file 42414_2024_135_MOESM1_ESM.docx]
